# A structured ICA-based process for removing auditory evoked potentials

**DOI:** 10.1038/s41598-022-05397-3

**Published:** 2022-01-26

**Authors:** Jessica M. Ross, Recep A. Ozdemir, Shu Jing Lian, Peter J. Fried, Eva M. Schmitt, Sharon K. Inouye, Alvaro Pascual-Leone, Mouhsin M. Shafi

**Affiliations:** 1 Berenson-Allen Center for Noninvasive Brain Stimulation, Beth Israel Deaconess Medical Center, 330 Brookline Avenue, KS-423, Boston, MA USA; 2 Department of Neurology, Harvard Medical School, Boston, MA USA; 3 Department of Medicine, Harvard Medical School, Boston, MA USA; 4 Hinda and Arthur Marcus Institute for Aging Research, and Deanna and Sidney Wolk Center for Memory Health, Hebrew SeniorLife, Boston, MA USA; 5 Guttmann Brain Health Institute, Institut Guttmann, Institut Universitari de Neurorehabilitació adscrit a la UAB, Badalona, Barcelona Spain

**Keywords:** Neuroscience, Physiology

## Abstract

Transcranial magnetic stimulation (TMS)-evoked potentials (TEPs), recorded using electroencephalography (EEG), reflect a combination of TMS-induced cortical activity and multi-sensory responses to TMS. The auditory evoked potential (AEP) is a high-amplitude sensory potential—evoked by the “click” sound produced by every TMS pulse—that can dominate the TEP and obscure observation of other neural components. The AEP is peripherally evoked and therefore should not be stimulation site specific. We address the problem of disentangling the peripherally evoked AEP of the TEP from components evoked by cortical stimulation and ask whether removal of AEP enables more accurate isolation of TEP. We hypothesized that isolation of the AEP using Independent Components Analysis (ICA) would reveal features that are stimulation site specific and unique individual features. In order to improve the effectiveness of ICA for removal of AEP from the TEP, and thus more clearly separate the transcranial-evoked and non-specific TMS-modulated potentials, we merged sham and active TMS datasets representing multiple stimulation conditions, removed the resulting AEP component, and evaluated performance across different sham protocols and clinical populations using reduction in Global and Local Mean Field Power (GMFP/LMFP) and cosine similarity analysis. We show that removing AEPs significantly reduced GMFP and LMFP in the post-stimulation TEP (14 to 400 ms), driven by time windows consistent with the N100 and P200 temporal characteristics of AEPs. Cosine similarity analysis supports that removing AEPs reduces TEP similarity between subjects and reduces TEP similarity between stimulation conditions. Similarity is reduced most in a mid-latency window consistent with the N100 time-course, but nevertheless remains high in this time window. Residual TEP in this window has a time-course and topography unique from AEPs, which follow-up exploratory analyses suggest could be a modulation in the alpha band that is not stimulation site specific but is unique to individual subject. We show, using two datasets and two implementations of sham, evidence in cortical topography, TEP time-course, GMFP/LMFP and cosine similarity analyses that this procedure is effective and conservative in removing the AEP from TEP, and may thus better isolate TMS-evoked activity. We show TEP remaining in early, mid and late latencies. The early response is site and subject specific. Later response may be consistent with TMS-modulated alpha activity that is not site specific but is unique to the individual. TEP remaining after removal of AEP is unique and can provide insight into TMS-evoked potentials and other modulated oscillatory dynamics.

## Introduction

Transcranial magnetic stimulation (TMS) has gained increasing importance and application in the neurophysiologic characterization of healthy aging^1,2^ and a variety of neurological disorders, including Alzheimer’s disease^1,2^, epilepsy^3^, autism spectrum disorders^2^, and schizophrenia (see^4^ for a review). TMS excites neuronal populations^5^ in cortical networks^6,7^. TMS-evoked^8^ and induced changes in endogenous oscillatory activity^9^ can be observed using electroencephalography (EEG)^10,11^. An averaged TMS-evoked EEG potential (TEP) is used to describe local and network effects of focal single pulse TMS^8,10,12,13^. However, the TEP waveform contains all activity within several hundred milliseconds of the pulse. This activity can include centrally evoked activity from induced intracranial currents, modulated oscillatory dynamics^9,14,15^, peripherally evoked activity from direct sensory afferents due to the multi-sensory nature of each TMS pulse^16–20^, and non-neural artifacts. Existing preprocessing pipelines are fairly good at isolating and removing non-neural artifacts but are inconsistent at separating centrally and peripherally evoked activity.

The TEP reflects TMS-evoked brain activation^21,22^ that can be specific to stimulation site^9,13,23^, and reproducible^13,23,24^, particularly at earlier latencies. However, the specificity of longer latency responses is a matter of debate^16,25,26^ due to high correlation between TEPs and sensory responses to TMS by 60–80 ms^17,18,23^ and high correlation between active and sham stimulation starting as early as 58 ms^16,18^. Biabani et al.^17^ raised concerns about the specificity of TEPs to TMS-evoked activity, advising a need to further suppress sensory evoked activity or to remove it.

One large sensory component appears to be due, at least in part, to auditory processing of the “click” caused by mechanical deflection of the copper coil when electrical current passes through it^27^. This auditory evoked potential (AEP) dominates the TEP waveform^16^ and is a stereotyped response to an auditory stimulus, with peaks at 50 ms from primary auditory cortical response and at 100 and 200 ms from responses in surrounding belt areas of A1^28–30^. Because AEP source is bilateral auditory cortex, the distribution of the response in scalp electrodes is central^30–32^. AEP might explain TEP non-specificity across site and stimulation conditions^16^.

Earplugs and noise masking^6,18,33^ can attenuate AEP in single pulse TMS-EEG, and foam padding between coil and scalp can reduce bone conduction of the sound^34^. Rocchi et al. showed that noise masking can reduce AEP effectively for subthreshold stimulation^18^. Use of these experimental modifications, along with adjustment of coil placement and stimulation parameters during data acquisition, can minimize sensory potentials and maximize the impact of TMS on cortex^26^. However, many groups have observed AEP even after using these techniques^16,17,34–36^. When masking needs to be presented at louder volumes, such as for suprathreshold stimulation, tolerability for the loud masking sound becomes a greater concern^16^; and if the noise-masking is painful, for pain induced evoked potentials and oscillatory changes with pain^37–39^. In some cases, participants can still hear the distinct sound of the TMS click through noise, regardless of volume. It has been suggested this could be due to contrasting acoustic properties of the TMS click and sustained noise^40^. Perhaps critical to the discussion, a sensory potential with identical time course and topography has been described with somatosensory^19^ and multimodal^41–43^ perception, so there may be contributions to the AEP that cannot be masked with an auditory sound. In addition, it is unknown what effects noise masking sounds have on the TEP. Auditory noise is known to have measurable impacts on sensory processing of other sounds^44^, sensorimotor cortical excitability^45–47^, cognitive task performance^48^, and on evoked potentials^49–51^, but noise effects on TEP are unexamined.

Because the sensory potential described as AEP is shared between active and sham TMS, sham could be useful for identifying this AEP in TEP. Rogasch et al.^52^ showed that Independent Components Analysis (ICA)^53,54^ can detect AEPs in TEPs from both active and sham TMS and suggested that concatenating active and sham recordings before ICA would reveal a common AEP potential in both conditions. Biabani et al.^17^ reported a correlation between TEPs from active TMS and TMS to the shoulder starting at latencies of 60 ms and used ICA on merged active and sham conditions to detect sensory potentials in a comparison with other methods. However, to our knowledge, this approach has not been rigorously examined or quantified in non-M1 targets and has not been evaluated using different populations and varying sham protocols.

In the current study, we merge sham and two active stimulation conditions before ICA to identify a common AEP component. Because of additional sensory components that occur with M1 stimulation, we used non-M1 stimulation sites for the active stimulation conditions. We quantify the changes that occur when removing AEP using this method and characterize the components that account for TEP non-specificity. This manuscript presents these four specific contributions: (1) We assess the generalizability of this approach by applying it to two populations: younger and older adults, collected with differing sham procedures and TMS-EEG systems. (2) We assess and quantify the impact of removing AEP with this method. (3) We quantify the specificity of the TEPs across different non-motor stimulation sites and between individuals before and after this procedure. Finally, (4) we describe the residual activity. Our hypothesis was that effectively removing AEP would reveal stimulation site-specific and participant-specific responses in early, mid latency and late time windows.

## Materials and methods

### Participants

Data used in the present analysis were from two ongoing TMS-EEG studies at the Berenson-Allen Center for Noninvasive Brain Stimulation at Beth Israel Deaconess Medical Center. All participants provided written informed consent before enrollment according to the Declaration of Helsinki. Both studies were approved by the Institutional Review Board of the Beth Israel Deaconess Medical Center, Boston, MA. The first cohort consists of 10 healthy adult control participants (9 M/1F, age = 42.2 ± 18.8 yrs., range = 19 to 65) from a TMS-EEG study of epilepsy^3^. The second cohort consisted of 24 older adults, collected as part of a study of postoperative delirium called The Successful Aging after Elective Surgery study (SAGES; 10 M/14F, age = 72.0 ± 6.6 yrs., range = 65 to 89)^55^. Participants selected for SAGES did not have clinical dementia, were scheduled for major surgery, and we used pre-surgery baseline TMS-EEG visit data for this analysis. We replicated and validated the findings from the first cohort (healthy younger adult) with the second cohort (older adult). The two cohorts represent different age groups, sham TMS protocols, and TMS-EEG systems, and are therefore ideal for addressing the robustness and strengths/weaknesses of this structured ICA-based approached to isolating AEP from TEP independent of experimental design.

### Transcranial magnetic brain stimulation set-up and procedure

In the younger cohort, TMS was delivered using a Nexstim eXimia stimulator with real-time MRI neuronavigation (NBS software v3.2.1; Nexstim, Helsinki, Finland), following standard protocols^56^. Single TMS pulses were applied to left dorsolateral prefrontal cortex (L DLPFC), left intra-parietal lobule (L IPL) and a sham condition to left M1 delivered with an active coil tilted away from the scalp at 90°. DLPFC and IPL were anatomically defined, respectively, as the superior half of the middle frontal gyrus approximately 3 cm anterior to precentral sulcus and the superior edge of angular gyrus roughly 1 cm inferior to intraparietal sulcus. Coil orientation was set with coil handle perpendicular to the targeted gyri inducing a biphasic anterior–posterior—posterior-to-anterior current in the underlying cortex. No auditory noise masking or electrical stimulation was used. Participants were asked to wear earplugs during sham and active-TMS trials to protect their hearing. Throughout the visit, participants were seated in an adjustable chair. The motor hotspot for eliciting motor evoked potentials (MEPs) in the right first dorsal interosseous (FDI) muscle was determined by delivering single pulse TMS to the hand knob of left motor cortex with the coil tangential to the scalp and oriented orthogonal to the motor strip (~ 45° from anterior–posterior midline) and searching around until finding the site where the largest and most consistent MEPs were produced in FDI. Resting motor threshold (RMT) was then determined as the minimum stimulation intensity (% of maximum stimulator output) needed to elicit MEPs ≥ 50 µV in the relaxed FDI in at least 5 out of 10 pulses. Following determination of RMT, 120 single pulses of TMS were applied at 120% RMT with randomly jittered inter-stimulus intervals (3–5 s). TMS operators monitored participants for wakefulness. See Vink et al.^57^ for more details.

In the older cohort, TMS was delivered using a figure-of-eight shaped coil with dynamic fluid cooling, a biphasic waveform, and anterior–posterior–posterior-anterior current direction in the brain (MagVenture Cool-B65) attached to a Magpro X100 stimulator, following standard protocols^56^. MagVenture Cool-B65 A/P coil was used with a 2.7 cm thick plastic spacer for sham stimulation and white noise masking was presented through earplug-earbuds at the maximum volume comfortable for each participant. Participants were asked to rate the perceived loudness of the TMS “click” while listening to the noise, relative to no noise-masking (0–10 scale, with 0 = “could not hear” and 10 = “as loud with noise and without noise”), and the average loudness rating was 6 ± 4. Four subjects reported “click” loudness ratings equal to 0. Although auditory noise masking was used in the older cohort, 20 participants reported hearing the TMS “click” and had AEPs. These 20 participants were used for all pre/post AEP removal comparisons (7M/13F, age = 71.9 ± 5.8 years, range = 65 to 86). Small current pulses (between 2 and 4 mA) were delivered over the left forehead, over the frontalis muscle, using surface electrodes (Ambu Neuroline 715) to approximate somatosensory sensations arising from skin mechanoreceptors and scalp muscles during the active-TMS condition. The motor hotspot for eliciting motor evoked potentials (MEPs) in the right FDI muscle was determined by delivering single pulse TMS to the hand knob of left motor cortex with the coil tangential to the scalp and oriented orthogonal to the central sulcus/motor strip (~ 45° from mid-sagittal plane) and varying the location and orientation until the largest and most consistent MEPs were produced in FDI. RMT was determined as the minimum stimulation intensity needed to elicit MEPs ≥ 50 µV in the relaxed FDI in at least 5 out of 10 trials. Following determination of the FDI hotspot and RMT, 120 single pulses of TMS were applied at 120% RMT with randomly jittered inter-stimulus intervals (3–5 s) to the DLPFC and IPL targets, and sham TMS over M1. To select the non-motor targets in the SAGES cohort, group-level resting-state functional networks maps were used based on a 7 networks parcellation covering cortical and subcortical structures^58^. Confidence maps for each RSN were used, representing the confidence of each vertex belonging to its assigned network across a sample of 1000 healthy participants (expressed as valued between − 1 and 1), with larger values indicating higher confidence. By using group-level functional parcellations and confidence maps on the Montreal Neurological Institute (MNI) template brain, we were able to target the most consistent and reliable regions within the left angular gyrus (L-IPL: − 55.1, − 70.5, 27.7) and dorsolateral prefrontal cortex (L-DLPFC: − 50.5, 30.4, 31.8) that had the highest likelihood of occurring in the default mode and frontoparietal resting-state networks, respectively. A custom processing pipeline was developed to take each participant’s anatomical MRI, create a non-linear transform from the participant’s native space to MNI space and then use the inverse of that transform to bring back the coordinates into participant’s space using FSL’s FNIRT tool. The transformed coordinates along with individual high-resolution T1w images were then imported into the Brainsight TMS Frameless Navigation system (Rogue Research Inc., Montreal, Canada), and co-registered to digitized anatomical landmarks for online monitoring of coil positioning. TMS operators monitored participants for wakefulness.

### Electroencephalography acquisition and preprocessing

In the younger cohort, whole scalp 60-channel EEG was collected with a TMS-compatible amplifier (eXimia EEG, Nexstim Ltd) and labeled according to the extended 10–20 international system. EEG data were online referenced to an additional electrode on the forehead. Impedances were maintained below 5 kΩ at a sampling rate of 1450 Hz. EEG signals were digitized using a Nexstim amplifier. Digitized EEG electrode locations on the scalp were also co-registered to individual MRI scans using Nexstim Brainsight TMS Frameless Navigation system (Rogue Research Inc., Montreal, Canada). See Vink et al.^57^ for more details.

In the older cohort, whole scalp 64-channel EEG was collected with a TMS-compatible amplifier (actiCHamp, Brain Products GmbH, Munich, Germany) and labeled according to the extended 10–20 international system. EEG data were online referenced to AFz. Impedances were maintained below 5kΩ at a sampling rate of 5000 Hz. EEG signals were digitized using a BrainVision amplifier (BrainCHamp, BrainVision Recorder software, v. 1.21). Digitized EEG electrode locations on the scalp were co-registered to individual MRI scans using Nexstim Brainsight TMS Frameless Navigation system (Rogue Research Inc., Montreal, Canada).

EEG data were pre-processed offline using custom scripts and EEGLab v2019.0^59^ in MATLAB R2019a (Mathworks, Natick, MA, USA). Data were segmented into 3000 ms epochs, each starting 1000 ms before (pre-stimulus) and ending 2000 ms (post-stimulus) following TMS pulse, respectively. Baseline correction was performed by subtracting the mean pre-stimulus (− 900 to − 100) signal amplitude from the rest of the epoch in each channel. Following baseline correction, data were visually inspected to identify and remove extremely noisy channels with high amplitudes. The median number of channels removed in the younger cohort was 2 channels (range: 0–6), and in the SAGES cohort was 1 channel (range: 0–4). Zero-padding between − 2 and 14 ms time range were then applied to remove the early TMS pulse artifact from the EEG data. Both cohorts were affected by muscle artifacts from the TMS, so we applied a standard zero-padding approach to equate analysis between the two datasets, and subsequently employed conventional ICA methods to identify and remove muscle artifacts. All zero padded epochs were then tagged based on voltage (≥ 100 μV), kurtosis (≥ 3), and joint probability (single channel-based threshold ≥ 3.5sd; all channel-based threshold ≥ 5sd) metrics to identify excessively noisy epochs. Visual inspection was performed on the tagged epochs for the final decision on removal of noisy epochs.

An initial round of fast independent component analysis (fICA)^60^ was performed to identify and remove exclusively components with early TMS evoked high amplitude electrode and EMG artifacts^52,61^. After the first round of fICA, the EEG data were interpolated for previously zero-padded time window around TMS pulse (− 2 ms to 14 ms) using linear interpolation, band pass filtered using a forward–backward 4th order Butterworth filter from 1 to 100 Hz, notch filtered between 57 and 63 Hz, and referenced to global average. Missing/removed channels were interpolated using spherical interpolation, and DLPFC, IPL and sham stimulation blocks were merged, in that order. Because recordings were made with 60/64 channels, and the signals were unlikely to have that many independent sources, PCA was used to reduce dimensionality prior to ICA. This approach can improve decomposition^62,63^ and signal to noise ratio of large sources^64^. Data were reduced into no fewer than 30 dimensions in the younger cohort (median = 32.5, range = 30–50), and no fewer than 35 dimensions in the older cohort (median = 45, range = 35–60), optimized at the individual level to maximize uniqueness and dipolarity of resulting components while minimizing residual noise components^64^. A second round of fICA was run on the merged data to manually remove all remaining artifact components^61^ including eye movement/blink, muscle noise (EMG), single electrode noise, TMS evoked muscle, and cardiac beats (EKG). The median number of components removed in the younger cohort was 21.5 (range: 18–41), and in the older cohort was 38.5 (range: 28–55). The data were low pass filtered with a 4th order Butterworth filter at 50 Hz.

Components that met the following criteria were classified as AEP components and removed: (1) the time-course has three peaks, P50-N100-P200, with the lowest amplitude in the P50 compared with N100 and P200; (2) the scalp topography reflects left/right symmetry and a central distribution anterior to Cz; and (3) the component is shared across both active stimulation sites and sham stimulation. The median number of remaining components after all cleaning steps in the younger cohort was 8.5 (range: 6–11), and in the older cohort was 7 (range 4–16). See “Discussion” for more details about AEP classification criteria. Conditions were then unmerged for subsequent analyses.

In both rounds of fICA, a semi-automated artifact detection algorithm incorporated into the open source TMS-EEG Signal Analyzer (TESA v1.1.0-beta) extension for EEGLAB was used to classify and visually inspect components based on their frequency, activity power spectrum, amplitude, scalp topography, and time course^65^ (http://nigelrogasch.github.io/TESA/).

### Analysis of TMS-evoked potentials

All analyses were performed at the single subject level, and group level statistical analyses were performed. Selections of both individual and group data are shown in several figures and supplementary figures. The specific analyses are described here, of time course and topography of TEPs, GMFP and LMFP, TEP similarity, source reconstruction, and follow-up exploratory analyses of remaining TEP.

#### Time-course and topography

Latency and amplitude of peaks in the pre- and post-AEP removal TMS-evoked cortical response were identified using an automated *peakfinder* algorithm^66^ and scalp topographies were plotted at those peak latencies.

#### GMFP/LMFP (and windows)

Global mean field power (GMFP) across all channels was used to quantify global synchrony and excitability^67,68^ in the 14–400 ms post-TMS window. We computed Global Mean Field Power (GMFP) following:

$$GMFP\left(t\right)=\sqrt{\left\{\frac{{\sum }_{i}^{k}(\mathrm{V}i (\mathrm{t})-{\mathrm{V}}_{mean(t)})^{2}}{K}\right\}}$$

where V_*i*_(t) is the voltage at electrode *i* at a certain point in time, V_mean(t)_ is the mean of instantaneous TEP across electrodes, and *K* is the number of electrodes.

Local mean field potentials (LMFP) from electrodes near the active stimulation sites were used to quantify local activity under the coil^67,69,70^ in the 14–400 ms post-TMS window. Channels included in each region of interest were determined as all electrodes bordering stimulation target locations. For the younger cohort, the DLPFC ROI included AF1, F7, F5, F1, FT7, FC5, and FC3 and the IPL ROI included TP7, CP5, CP3, P7, P3, and PO3. In the older cohort, the DLPFC ROI included AF3, F7, F5, F3, FT7, FC5, and FC3, and the IPL ROI included TP7, CP5, CP3, P7, P5, P3, PO7, and PO3. See Supplementary Fig. S1 for electrode arrays color coded to show LMFP ROIs for younger adult (S1A) and older SAGES (S1B) cohorts. In addition, GMFP and LMFP were calculated for three windows corresponding to the three subcomponents of AEP. Windows were defined in each study cohort for GMFP, LMFP of the DLPFC ROI, and LMFP of the IPL ROI, using the latency of the minimum amplitude between peaks in the sham stimulation condition (determined using the *peakfinder*() function, which finds peaks with amplitudes that are greater than two standard deviations above baseline^66^). See Fig. 2D for time-windows defined for GMFP analysis in the younger cohort, and Supplementary Fig. S2 for all defined windows in both cohorts. Area under the curve for GMFP, LMFP at the DLPFC ROI, LMFP at the IPL ROI, and in all time-windows were entered as dependent variables into a 2 (*Version*: pre-AEP removal, post-AEP removal) by 3 (*Site*: DLPFC, IPL, shamM1) repeated-measures analyses of variance (ANOVA).

#### Similarity index

Similarity between TEPs across site and between subjects at each site, pre and post AEP removal, was quantified in vector space using the cosine similarity index (SI) which ranges from 0 to 1 (low to high similarity, with 0.5 indicating mid-range)^13,23^. We first generated a TEP matrix for each subject (from averaged responses) with a fixed window size (385 ms) covering EEG responses from 14 to 400 ms. Each TEP matrix contains millisecond voltage values from all channels with a 63 × 385 matrix size. We then used cosine similarity to quantify similarity index (SI) between matrices following:

$${SI}_{XY}=\frac{\sum_{i,t=1}^{n}{(X}_{it}*{Y}_{it})}{\sqrt{(\sum_{i,t=1}^{n}{X}_{it}^{2}})*(\sum_{i,t=1}^{n}{Y}_{it}^{2})}$$

where ${SI}_{XY}$ is the cosine similarity between TEP matrices x and y for a given stimulation site, and *n* is the number of channels, *X*_*it*_ and *Y*_*it*_ are the *i*th vector of all channels at time *t*. Within-subject site SI was computed as the mean SI from the diagonal of site comparison grids. Between-subject SI was computed as the mean SI of half grid, excluding the diagonal, of within-site grids (DLPFC by DLPFC, etc.). To enable assessment of similarity between the DLPFC and IPL datasets, SI was also calculated for three time-windows defined by residual TEP in active stimulation conditions post-AEP removal: early, mid, and late latency. These windows were defined using GMFP area under the curve post-AEP removal active stimulation conditions averaged across participants.

We performed multiple univariate tests to describe how the AEP-removal method affects each outcome variable separately. Multiple univariate tests are preferred over one multivariate MANOVA here for a number of reasons^71^. First, our research question dictates a model that tests for effects of the method on each outcome variable independently, with no particular interest in a linear composite of the outcome variables. Our approach best describes how using this AEP-removal method will practically influence a variety of datasets if applied. We acknowledge that redundant information is being obtained using multiple ANOVAs but suggest that this redundancy supports that removing AEP using this method has robust effects on mean field potentials across ROIs and, particularly at mid and later latencies. However, to address concerns about experiment-wise Type 1 error for performing multiple ANOVAs, we additionally use an additive Bonferroni inequality. For *m* = 12 tests, α of 0.05 becomes 0.004 (0.05/12).

#### EEG source reconstruction

All TMS evoked EEG source reconstruction was performed using Brainstorm^72^. First, digitized EEG channel locations and anatomical landmarks of each subject were extracted from Brainsight (nasion ‘NAS’, left pre-auricular ‘LPA’, and right pre-auricular ‘RPA’ points; Rogue Research Inc., Montreal, Canada), and registered onto individual MRI scans in brainstorm. Next, the EEG epochs, − 500 ms to 1000 ms with respect to TMS pulse for each TMS trial were uploaded, and the average epoch time series was generated for each subject. Forward modeling of neuro electric fields was performed using the open MEEG symmetric boundary element method, all with default parameter settings. Noise covariance was estimated from individual trials using the pre-TMS (− 500 to − 1 ms) time window as baseline. The inverse modeling of the cortical sources was performed using the minimum norm estimation (MNE) method with dynamic statistical parametric mapping (dSPM) and constraining source dipoles to the cortical surface. The resulting output of EEG source reconstruction was the MNE current density time series for each cortical vertex.

#### Residual mid-latency components

As a follow-up we examined the residual TEP in a mid-latency window. We used two approaches in an attempt to isolate the mid-latency components in sham—(1) a percent variance threshold to identify components contributing the most variance in this mid-latency window, and (2) non-parametric permutation based suprathreshold cluster size analysis^73^. We looked for clusters in the post pulse GMFP that were greater than the baseline period, and we determined which components contributed the highest percent variance to each of these clusters. See “Results”—“Residual mid latency components” for more details. Due to redundancy in the results of these two approaches, only the percent variance threshold approach (1) is described here. See Supplementary material for the results of the non-parametric permutation-based cluster size analysis (2).

All plots were generated using custom scripts and MATLAB R2019a (Mathworks, Natick, MA, USA), and figures were designed using Inkscape (version 1.1, http://www.inkscape.org/).

## Results

**Figure 1:**
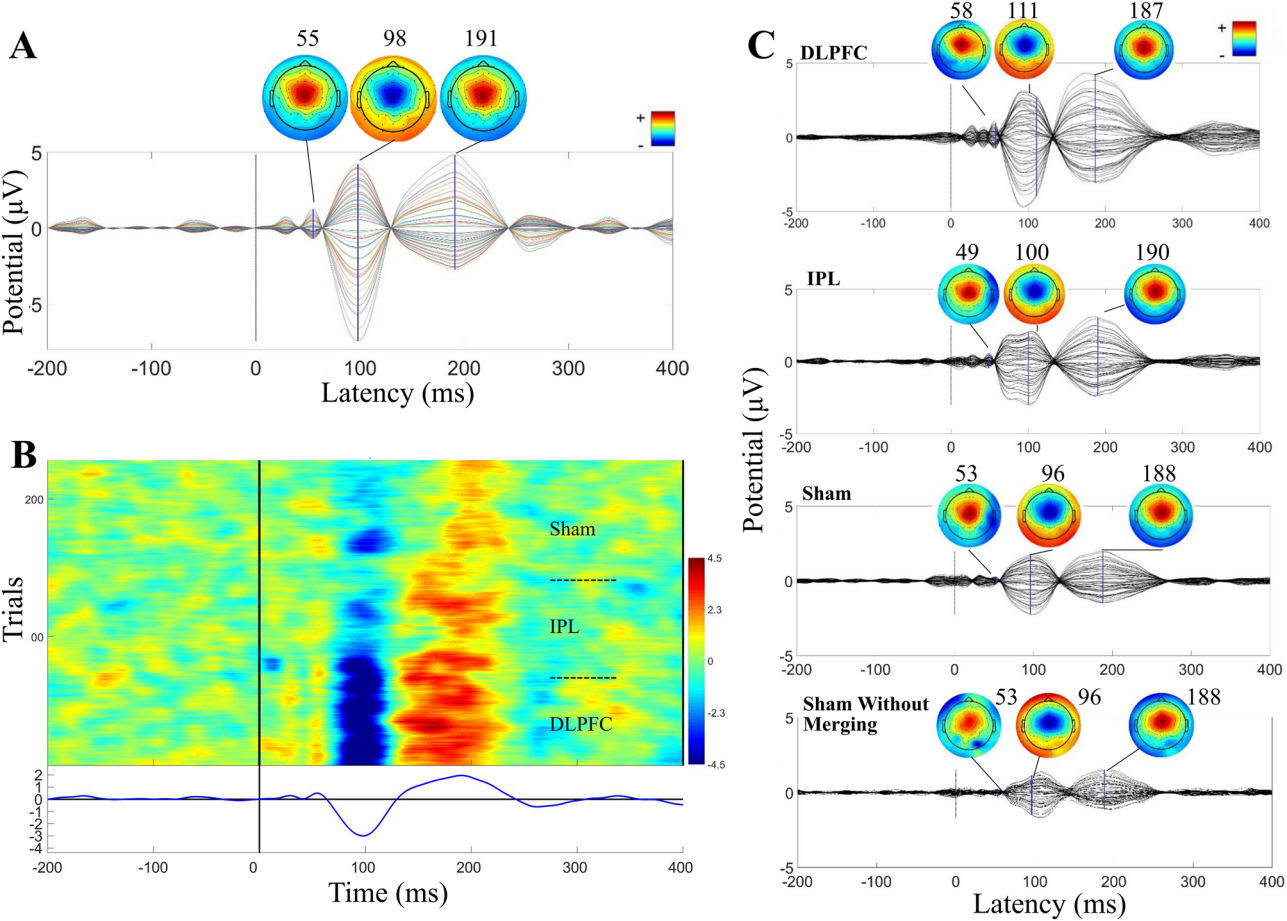
Auditory evoked potential (AEP) in the younger adult cohort. (**A**) Time-course and topography of AEP component removed from merged dataset in a representative participant. (**B**) ERP Image of AEP component in merged dataset across all trials in the same representative participant. Conditions were merged in this order: DLPFC, IPL, sham. (**C**) Components removed, averaged across all participants (N = 10; after being unmerged into conditions), and components removed from sham without merging with other conditions, averaged across all participants (N = 10).

**Figure 2:**
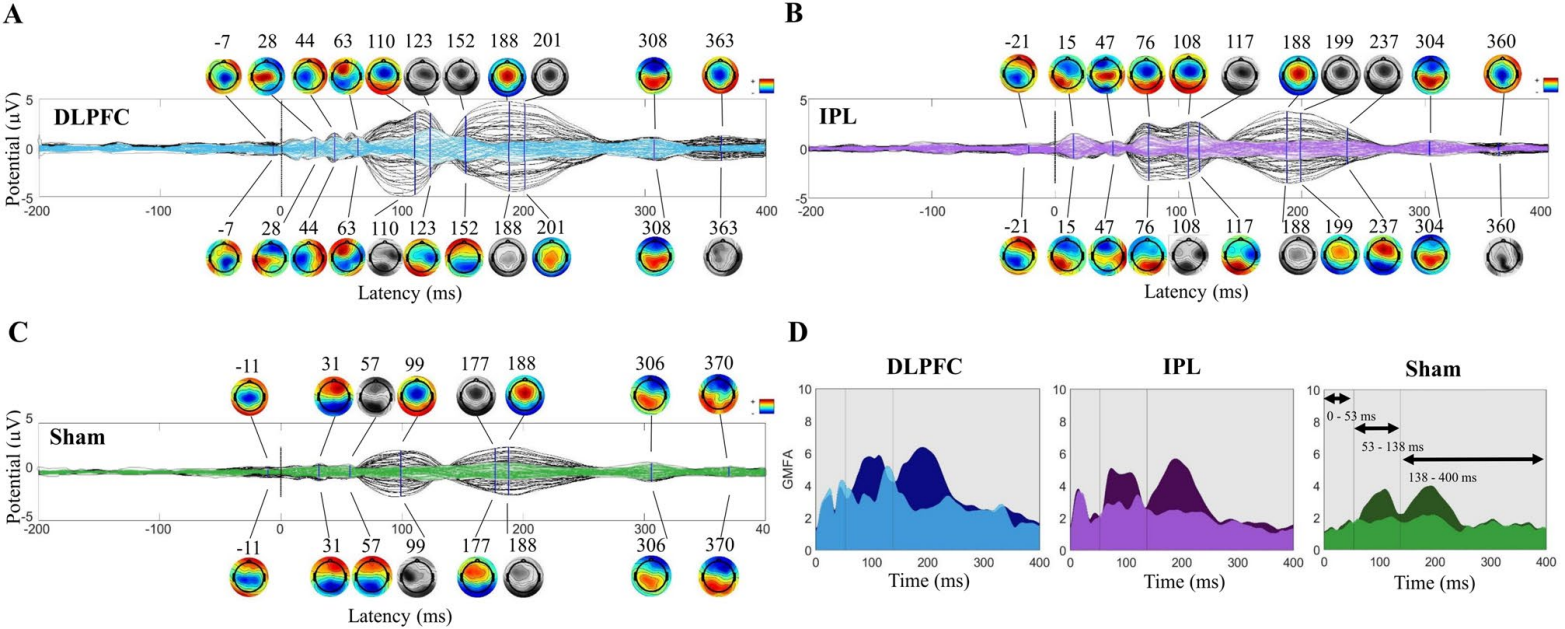
Group level impact of removing AEP in the younger cohort (N = 10). Topoplots are greyscale if no peak is present at the indicated latency. (**A**) DLPFC condition TEPs pre (black) and post (blue) removal of AEP components. Scalp topography pre (above) and post (below) at indicated latencies. (**B**) IPL condition TEPs/topographies pre (black/above) and post (purple/below) removal of AEP components. (**C**) Sham stimulation condition TEPs/topographies pre (black/above) and post (green/below) removal of AEP components. (**D**) Global mean field potential (GMFP) area under the curve plots pre (darker) and post (lighter) AEP removal in three time-windows, defined by sham GMFP peak activity pre-AEP removal.

**Figure 3:**
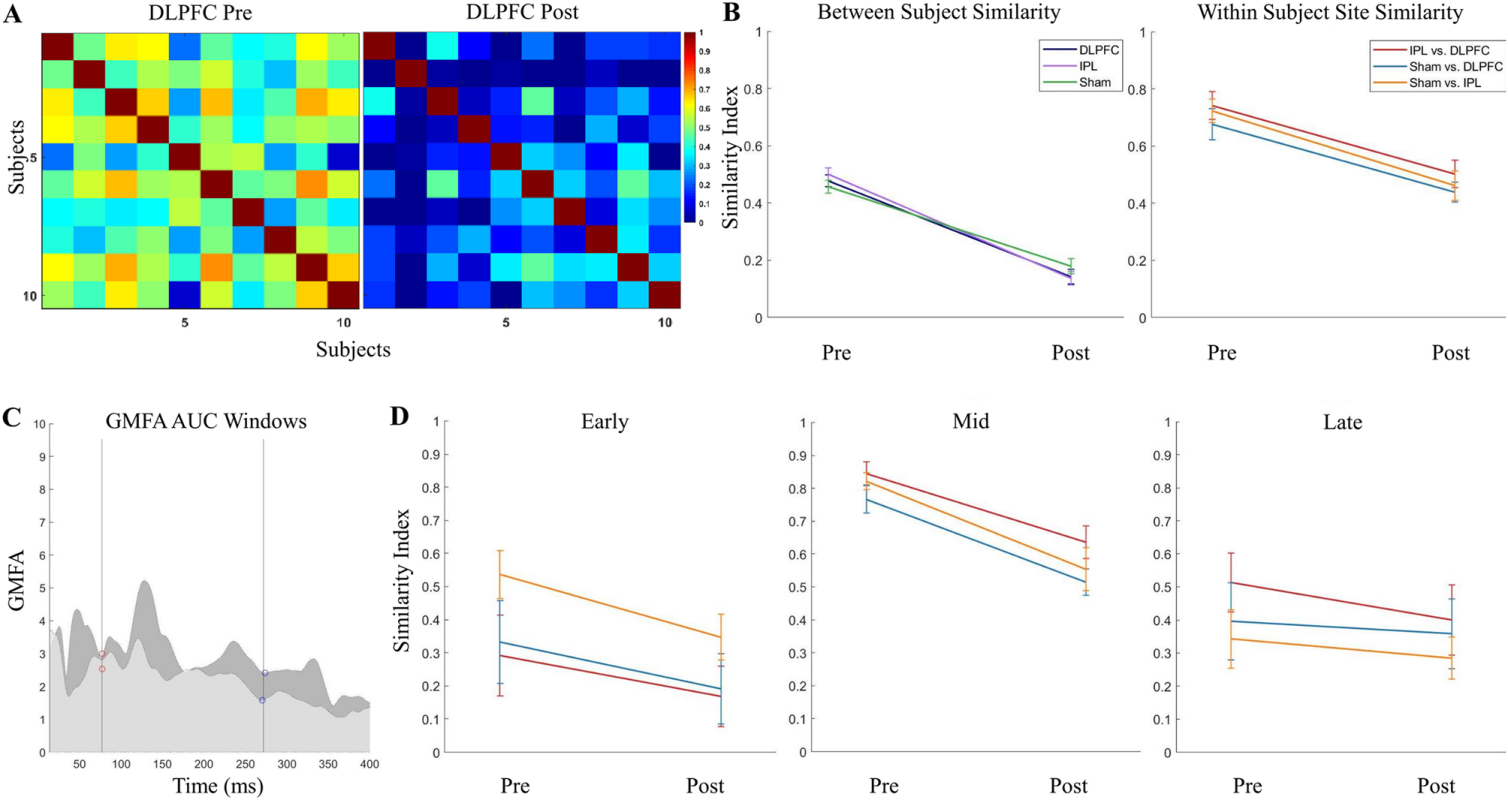
Specificity of TEPs (cosine similarity) before and after removing AEPs in the younger cohort. (**A**) Between-subject similarity index grids pre- and post-removal of AEP components. (**B**) Between-subject similarity index average (calculated from half of the grid, excluding the diagonal) pre- and post-removal (left), and within-subject stimulation site similarity (calculated as the average of the diagonal of IPL/DLPFC, sham/DLPFC, and sham/IPL grids; right). (**C**) Time windows used for within-subject site similarity in three windows (early: 14–77.24 ms, mid: 77.24–271.72 ms, late: 271.72–400 ms), defined using the post-removal GMFP from DLPFC (darker) and IPL (lighter). (**D**) Within-subject stimulation site similarity pre and post removal in early, mid and late latency windows.

**Figure 4:**
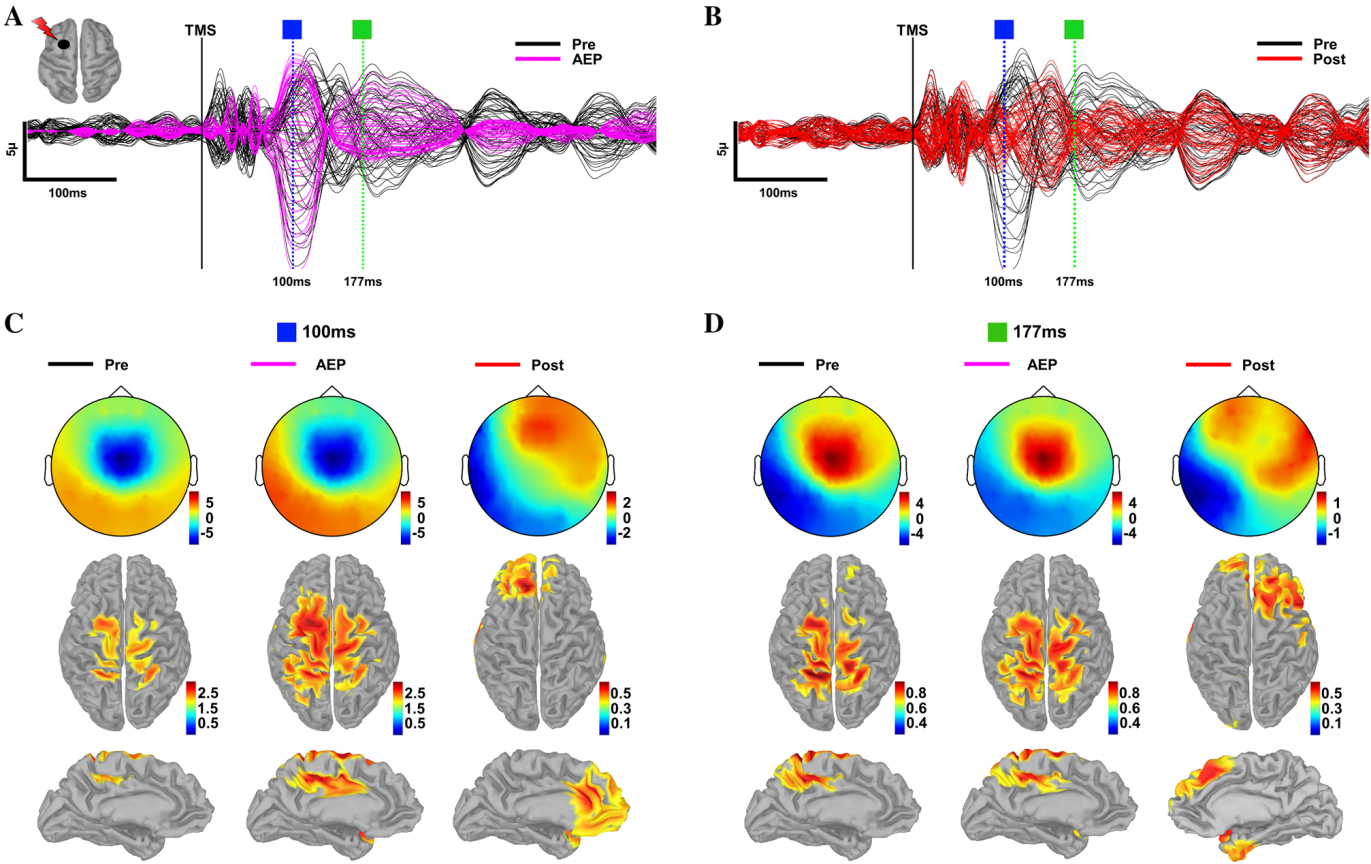
Source reconstruction of TEPs pre- and post-AEP removal. (**A**) TEPs from DLPFC pre- (black) AEP removal and AEP component (magenta) time series for a representative subject with selected peaks. (**B**) TEPs in (**A**) pre- (black) and post-(red) AEP removal for this same subject with selected peaks. (**C**) Topography and corresponding source reconstructions at 100 ms for pre- (left), AEP (middle) and post- (right) AEP removal. (**D**) Topography and corresponding source reconstructions at 177 ms for pre- (left), AEP (middle) and post- (right) AEP removal.

**Figure 5:**
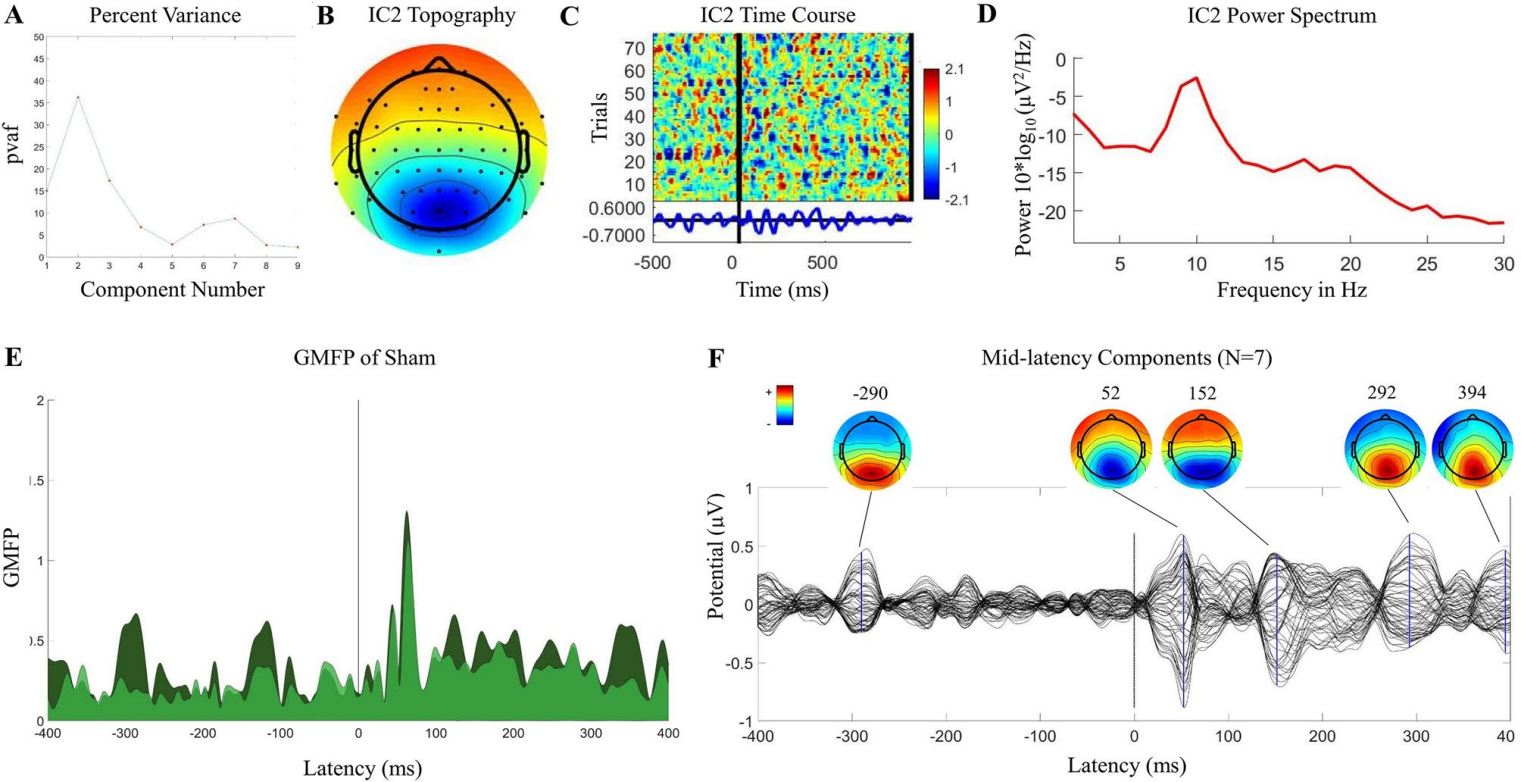
Mid-latency components. Properties of mid latency component in a one representative participant in sham stimulation condition (**A**–**E**). (**A**) Percent variance contributing to mid latency window in sham stimulation in all components, showing peak contribution in component 2. For all participants, a threshold PVAF of 28% was used to select largest mid-latency contributors in sham stimulation condition. (**B**) Topography of component 2, showing posterior midline projection. (**C**) ERP Image of component 2, showing time course across all trails and presence of activity in baseline period. (**D**) Spectral profile of component 2, with peak frequency in alpha band. (**E**) GMFP pre (darker) and post (lighter) removal of component 2, showing similar reduction in baseline and post-TMS windows. (**F**) TEP/topography of all mid latency components contributing more than 28% variance in mid latency window in sham stimulation condition (N = 7) from full group analysis.

**Figure 6:**
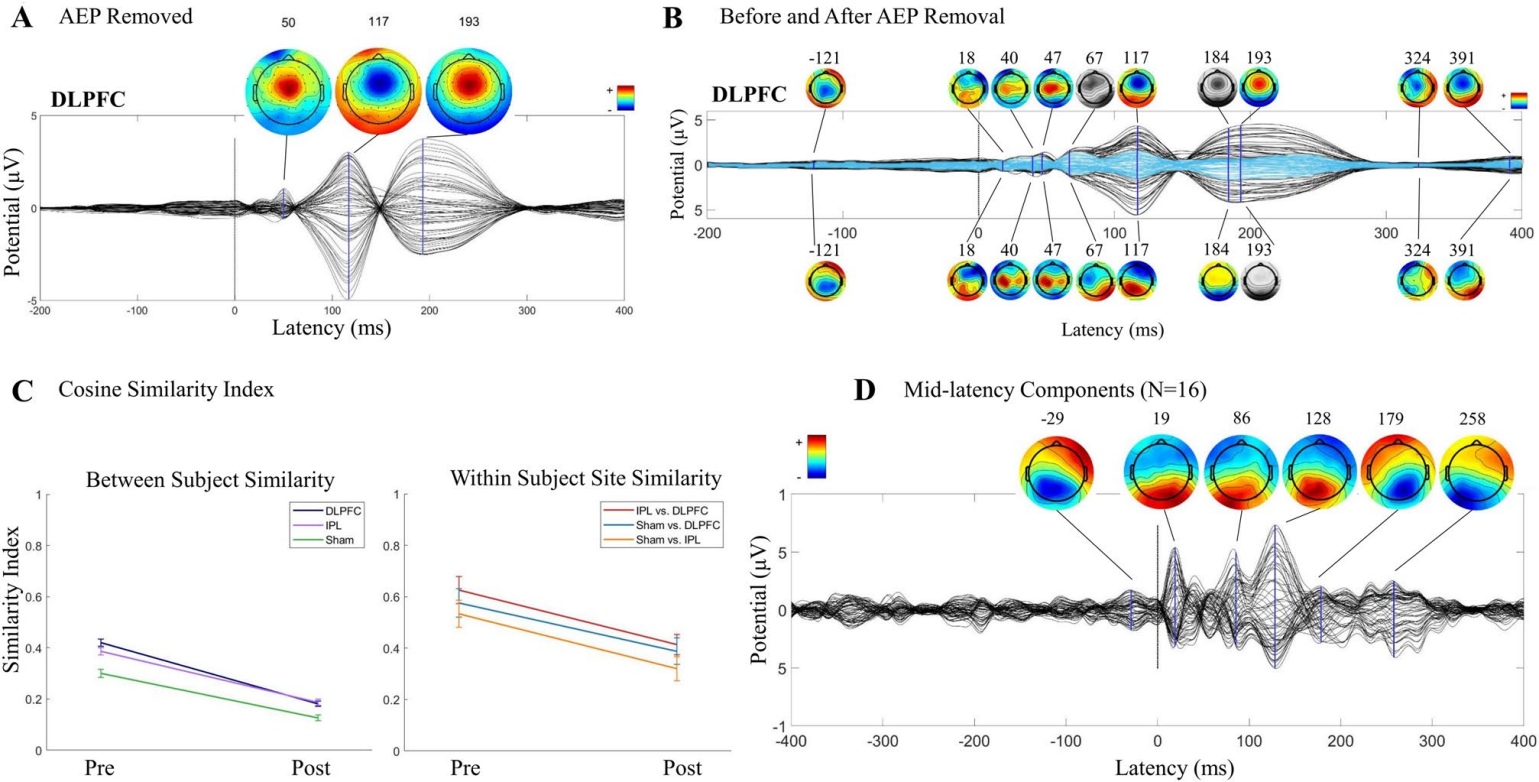
Validation using cohort of older adults (≥ 65 years) from SAGES study. 20 of the 24 participants have AEP components, regardless of the noise masking protocol, and this N = 20 is shown here. (**A**) AEP components removed, averaged across all participants (N = 20; DLPFC after being unmerged from other conditions). (**B**) DLPFC condition TEPs/topography pre (black/above) and post (blue/below) removal of AEP components. Topoplots are greyscale if no peak is present at the indicated latency. (**C**) Cosine similarity index average between-subject (left; calculated from half of the grid, excluding the diagonal) and within-subject site (right; calculated as the average of the diagonal of IPL/DLPFC, sham/DLPFC, and sham/IPL grids) pre and post removal of AEP components. (**D**) TEP/topography of all mid latency components contributing more than 28% variance in mid latency window in sham stimulation condition (N = 16) from full group analysis.

Results of all analyses are shown for the younger cohort in Figs. 1, 2, 3,4, 5, and summarized results are shown for the older cohort in Fig. 6. See Supplementary for additional Tables and Figures for both cohorts.

### The AEP

All 10 participants in the younger cohort had an AEP that met all three criteria (time-course, topography, and shared across conditions including sham). See Fig. 1A for a representative participant’s TEP and scalp topography of the component classified as AEP in this participant’s data. The ERP image of this component from the merged conditions shows a shared time-course and polarity of AEP across active and sham stimulation conditions (Fig. 1B for same representative participant). Averaged TEP and topography plots across all AEP components from the full cohort show the expected stereotypical time-course and topography (Fig. 1C). At the group level, AEP components (14–400 ms) showed low within-subject site-specificity with an averaged between-site SI of 0.83 ± 0.04. The AEP components showed moderate subject specificity, with an averaged within-site between-subject SI of 0.49 ± 0.03. For all between- and within-site SIs, see Supplementary Table S1.

In the older cohort, 20 of 24 participants had an AEP that met all three criteria. AEP components (14–400 ms) again had high similarity across sites, with an averaged within-subject between-site SI of 0.66 ± 0.06. Between-subject similarity was again moderate, with an averaged within-site between-subject SI of 0.36 ± 0.02. For all between- and within-site SIs, see Supplementary Table S1. See Fig. 6A for the averaged AEP component time-course and topography from active stimulation of the DLPFC target in the older cohort.

### Effects on the TEP

#### Time-course and topography

Pre/post AEP removal comparisons at the group level revealed an amplitude reduction that was most noticeable at approximately 100 and 200 ms latencies. See Fig. 2A–C for TEPs and scalp topographies before and after AEP removal in the first cohort (N = 10). TEP from stimulation of DLPFC (Fig. 2A) had peaks at 28, 44, 63, 110, 188, 308, and 363 ms pre-AEP removal, and AEP removal revealed smaller peaks at 123 and 152 ms. See Fig. 2B IPL stimulation at 15, 47, 76, 108, 188, 304, and 360 ms pre-AEP removal and the revealed peak at 117 ms post-AEP removal, and Fig. 2C sham stimulation at 31, 99, 188, 306, and 370 ms pre-AEP removal and the revealed peaks at 57 and 177 ms post-AEP removal. Scalp topographies in this mid-latency window shifted to posterior distributions with removal of AEP. Earlier peaks appeared to be stimulation site specific and have unique topographies, less effected by removal of AEP than later peaks.

In the older cohort, with removal of AEP there was a similar amplitude reduction in mid-latency peaks of the TEPs and revealing of smaller peaks in that window with a posterior shift in topographies. See Fig. 6B for pre- and post-AEP removal TEPs and scalp topographies in the SAGES cohort with DLPFC stimulation (note pre/post changes at 117 and 193 ms). See Supplementary Fig. S3 for sham condition.

#### Global mean field potentials

In the younger cohort, in the full 14–400 ms time window, across all electrodes, we found that removing AEP reduced GMFP area under the curve, (F(1,9) = 21.78, p = 0.001), with no significant version (pre/post) by site interaction (F(2,18) = 2.28, p = 0.13).

Using the peak/trough analysis of GMFP in the sham stimulation condition pre-AEP removal, we found three peaks that were greater than two standard deviations above baseline with latencies at 45 ms, 110 ms, and 192 ms (windows for analysis: 14 to 53 ms, 53 to 138 ms, and 138 to 400 ms; Supplementary Fig. S2A). In the P50 window, there was no change in GMFP AUC with removal of AEP (F(1,9) < 0.0001, p = 0.99), and no significant version (pre/post) by stimulation site interaction (F(2,18) = 1.41, p = 0.27). In the N100 window, there was a significant reduction in GMFP AUC (F(1,9) = 9.14, p = 0.014, but no version by site interaction (F(2,18) = 0.16, p = 0.86). In the P200 window, there was a significant reduction in GMFP AUC (F(1,9) = 20.49, p = 0.001), and also a significant version by site interaction (F(2,18) = 5.65, p = 0.012). See Fig. 2D for GMFP AUC pre- and post-AEP removal. See Supplementary Table S2 for all test statistics.

In the older cohort, in the full 14–400 ms time window, we found that removing AEP reduces GMFP AUC (F(1,19) = 29.27, p < 0.0001), with a *Version*-by-*Site* interaction (F(2,38) = 6.04, p = 0.005). Three peaks were detected at 43, 122, and 205 ms (windows: 14 to 59 ms, 59 to 163 ms, and 163 to 400 ms; Supplementary Fig. S2D). In the P50 window, there was no change in GMFP AUC (F(1,19) = 1.18, p = 0.29), and no interaction with site (F(2,38) = 0.41, p = 0.66). In the N100 window, there was a significant reduction in GMFP AUC (F(1,19) = 21.08, p < 0.0001), with an interaction between version and site (F(2,38) = 6.63, p = 0.003). In the P200 window, there was also a reduction in GMFP AUC (F(1,19) = 31.26, p < 0.0001), and an interaction with site (F(2,38) = 4.58, p = 0.017).

#### Local mean field potentials

In the younger cohort, local mean field potentials (LMFP) over DLPFC and IPL targets reveal a similar pattern of results as in GMFP. We found a reduction in LMFP AUC with removal of AEP (DLPFC ROI: F(1,9) = 13.41, p = 0.005; IPL ROI: F(1,9) = 27.70, p = 0.001), with no interaction between *Version* and *Site* (DLPFC ROI: F(2,18) = 1.73, p = 0.21; IPL ROI: F(2,18) = 2.46, p = 0.11).

Using the peak/trough analysis, we found windows defined for the DLPFC ROI as 14 to 54 ms, 54 to 146 ms, and 146 to 400 ms, and for the IPL ROI as 14 to 52 ms, 52 to 144 ms, and 144 to 400 ms (Supplementary Fig. S2B,C). Removing AEP did not reduce LMFP area under the curve in the P50 window in either ROI (DLPFC ROI: F(1,9) = 0.47, p = 0.51; IPL ROI: F(1,9) = 0.09, p = 0.77), with no *Version*-by-*Site* interactions (DLPFC ROI: F(2,18) = 2.57, p = 0.10; IPL ROI: F(2,18) = 0.31, p = 0.74). Removing AEP reduced LMFP AUC in the N100 window in the DLPFC ROI (F(1,9) = 6.61, p = 0.03) but did not reach significance in the IPL ROI (F(1,9) = 4.58, p = 0.061), and there were no *Version*-by-*Site* interactions (DLPFC ROI: F(2,18) = 0.74, p = 0.49; IPL ROI: F(2,18) = 0.041, p = 0.96). In the P200 window, there was a reduction in LMFP AUC (DLPFC ROI: F(1,9) = 17.93, p = 0.002; IPL ROI: F(1,9) = 26.69, p = 0.001), a *Version*-by-*Site* interaction in the IPL ROI (F(2,18) = 5.92, p = 0.011) but not in the DLPFC ROI (F(2,18) = 2.73, p = 0.092).

In the older cohort, we also saw a reduction in LMFP AUC with removal of AEP (DLPFC ROI: F(1,19) = 18.34, p < 0.0001; IPL ROI: F(1,19) = 16.89, p = 0.001), and in this cohort we found a *Version*-by-*Site* interaction (DLPFC ROI: F(2,38) = 5.98, p = 0.006; IPL ROI: F(2,38) = 4.55, p = 0.017). Three windows were defined for the DLPFC ROI as 14 to 69, 69 to 165, and 165 to 400 ms, and for the IPL ROI as 14 to 60, 60 to 165, and 165 to 400 ms (Supplementary Fig. S2E,F). Removal of AEP did not reduce LMFP AUC in the P50 window (DLPFC ROI: F(1,19) = 0.014, p = 0.91; IPL ROI: F(1,19) = 1.08, p = 0.31), with no *Version*-by-*Site* interactions (DLPFC ROI: F(2,38) = 0.42, p = 0.66; IPL ROI: F(2,38) = 0.029, p = 0.97). The reduction in LMFP with removal of AEP was being driven by the N100 (DLPFC ROI: F(1,19) = 19.50, p < 0.0001; IPL ROI: F(1,19) = 16.68, p = 0.001) and P200 windows (DLPFC ROI: F(1,19) = 15.42, p = 0.001; IPL ROI: F(1,19) = 13.49, p = 0.002). There were *Version*-by-*Site* interactions in this cohort in the DLPFC ROI (N100: F(2,38) = 5.37, p = 0.009; P200: F(2,38) = 3.32, p = 0.047) and the IPL ROI (N100: F(2,38) = 4.41, p = 0.019; P200: not significant F(2,38) = 2.73, p = 0.078).

To address concerns about experiment-wise Type 1 error for performing multiple ANOVAs, we additionally use an additive Bonferroni inequality. As described in “Materials and methods”, for *m* = 12 tests, α of 0.05 becomes 0.004 (0.05/12). Our general pattern of results remains significant across measures and cohorts, showing a reduction in GMFP and LMFP AUC for full TEP, and with most robust changes in the N100 and P200 latency windows. Interactions between version and site do not remain significant for most outcome measures, excepting GMFP of N100 in the older cohort.

See Supplementary Table S2 for results of all GMFP and LMFP analyses, with test statistics, using α of 0.01, 0.05, and 0.004.

#### Similarity index

In the younger cohort, in full TEPs (14–400 ms), we found a reduction in between-subject SI pre- (0.48 ± 0.012) to post- (0.15 ± 0.013) AEP removal (t(2) = 17.82, p < 0.0001), indicating increasing subject specificity within stimulation site with removal of AEP. We found a reduction in between-site SI pre- (0.71 ± 0.019) to post- (0.47 ± 0.018) AEP removal (t(2) = 9.17, p < 0.0001), indicating an increase in site specificity with removal of AEP, although it should be noted that 0.47 ± 0.018 was still moderately similar across site. See Fig. 3A,B for between-subject pre and post AEP removal grids (A) and between-subject and between-site SI averages pre and post AEP removal (B).

In the active stimulation conditions, GMFP post AEP removal appeared to have three latency windows, separated by troughs at 77.24 and 273.79 ms in DLPFC and 77.24 and 270.34 ms in IPL, with trough latencies averaged across active sites at 77.24 and 271.72 ms. These windows were chosen to enable assessment of similarity in residual TEP (post AEP removal) and were defined using *peakfinder*()^66^ as early: 14 to 77.24 ms, mid: 77.24 to 271.72 ms, and late: 271.72 to 400 ms (Fig. 3C). In the early window, between-site SI was 0.39 ± 0.076 pre-AEP removal, indicating site specificity, and 0.24 ± 0.056 post-AEP removal with no significant change from pre- to post-AEP removal (t(2) = 1.61, p = 0.092). In the mid-latency window, between-site SI was 0.81 ± 0.023 pre-AEP removal, indicating high similarity across sites in this window, and reduced to 0.57 ± 0.036 post-AEP removal (t(2) = 5.68, p = 0.0024). In the late window, between-site SI was 0.42 ± 0.05 pre-AEP removal and 0.35 ± 0.034 post-AEP removal with no significant change from pre- to post-AEP removal (t(2) = 1.15, p = 0.16). See Fig. 3D for between-site SI averages pre and post AEP removal in the three latency windows.

See Supplementary Table S3 for mean between-subject and within-subject between-site SIs for all stimulation conditions.

Similarly, in the older cohort, in full TEPs (14–400 ms), we found a reduction in between-subject SI pre- (0.37 ± 0.036) to post- (0.16 ± 0.019) AEP removal (t(2) = 5.03, p = 0.0037), and a reduction in between-site SI pre- (0.58 ± 0.027) to post- (0.37 ± 0.028) AEP removal (t(2) = 5.29, p = 0.0031). As described above, AEP components (14-400 ms) had an averaged between-subject SI of 0.36 ± 0.02 and an averaged between-site SI of 0.66 ± 0.06—the almost identical SIs of AEP components and of pre-AEP removal TEP (but reduced in post-AEP removal TEP) indicates that the AEP components are dominating the signal-to-noise ratio of the evoked response prior to removal.

In the active stimulation conditions, GMFP post AEP removal showed three latency windows, separated by trough latencies averaged across active sites at 86.80 and 285.20 ms. The three latency windows were defined using *peakfinder*()^66^ as early: 14 to 86.80 ms, mid: 86.80 to 285.20 ms, and late: 285.20 to 400 ms. In the early window, between-site SI was 0.27 ± 0.021 pre-AEP removal and remained low, 0.19 ± 0.038, post-AEP removal (t(2) = 1.98, p = 0.060). In the mid-latency window, between-site SI reduced from 0.72 ± 0.030 to 0.51 ± 0.040 pre- to post-AEP removal (t(2) = 4.17, p = 0.0070). In the late window, between-site SI was 0.24 ± 0.017 pre-AEP removal and 0.21 ± 0.020 with no change pre- to post-AEP removal (t(2) = 1.00, p = 0.19). See Fig. 6C for between-subject and between-site SI averages pre and post AEP removal in the older cohort, and Supplementary Table S3 for mean between-subject and within-subject between-site SIs for all stimulation conditions.

### Source analysis

When AEP is present, topographical distribution of selected peaks in TEPs in both datasets have similar spatial characteristics compatible with the signal morphology and topography of AEPs reported in the recent TMS-EEG and EEG literature^52,74–76^. While the presence of AEPs resulted in potentials with a uniform spatial topography and source activations at the expected time points, site-specificity of the evoked response and distinct source activations became clearly visible after removing AEP. See Fig. 4 for spatial and temporal characteristics of TEPs pre- and post-AEP removal for a representative subject.

### Residual mid latency components

As an exploratory analysis, we examined the residual TEP that exhibits moderately low site specificity in the mid latency window. Although between-site similarity was reduced in this mid latency window, it was still higher than the midpoint of SI range (0 to 1), at 0.57 ± 0.036 in the younger cohort and 0.51 ± 0.040 in the older cohort, indicating some residual similarity across stimulation sites. One possibility is that the AEP was not being fully removed. However, the altered TEP time course and posterior shift in scalp topography in this mid latency window (Figs. 2A–C and 6B) suggest that removing AEP revealed smaller amplitude components that are unique from AEP. We describe the residual TEP but do not make strong claims as to the specific nature of the activity. However, the residual mid latency TEP may be brain-related and unique from AEP.

To characterize the mid latency components in sham, we used a percent variance threshold to identify components contributing the most variance in this mid latency window. We looked for clusters in the post pulse GMFP that were greater than the baseline period, and we determined which components contributed the highest percent variance to each of these clusters.

#### Percent variance threshold for components that contributed most to the mid latency window

Percent variance (PVAF) was calculated for the mid latency window for each participant. See Fig. 5A for PVAFs of all components contributing to the mid latency window in one representative participant. In most participants, we found 1–2 components with a peak PVAF above 28%. This 28% threshold was selected using a data driven approach to isolating sources contributing most to the mid latency window. The representative participant shown in Fig. 5 had a peak PVAF in component 2. The scalp topography of IC2 (Fig. 5B) had a posterior distribution characteristic of occipital alpha. The time-course of IC2 in all trials (Fig. 5C) showed presence in baseline, increase in amplitude after the TMS pulse, and oscillations at approximately 10 Hz (Fig. 5C,D). After removing IC2, GMFP of the sham condition, calculated across all channels, decreased in both the pre- and post-stimulation windows (Fig. 5E). All peak PVAF components across participants that exceed 28% variance (N = 7) showed activity in the pre-TMS baseline window, highest power oscillation in the alpha band, and GMFP reduction when removed in both the pre- and post-TMS windows. See Supplementary Fig. S4A for individual subject component topographies that exceeded 28% in this mid latency window. All these components across participants had a posterior distribution in the scalp topography, although some were at the midline and some suggested left/right laterality. See Fig. 5F for the average TEP and topographies of all peak PVAF components that met the 28% threshold. Time-courses and topographies of the mid-latency components in sham suggested brain components that did not meet our time course/topography criteria for AEP. It is possible the components could be occipital alpha with modulated power in the post-TMS window.

Between-subject and within-subject site similarity SI was calculated for the identified mid-latency components. Within-subject between-site SI was 0.53 ± 0.039, and between-subject SI was 0.12 ± 0.034 for these components. Together, these SI values indicated that the mid latency components did not exhibit site specificity but did exhibit subject specificity. Time course of component amplitude peaks showed no consistency across site within-subject, suggesting these components were not time locked to the TMS pulse.

Similarly, in the older cohort, we identified peak PVAF contributors in mid latency sham using the same 28% variance threshold. 16 participants had at least one component that met this threshold. See Fig. 6D for the average TEP and topographies. These components exhibited posterior, either central or left/right lateral, scalp distributions, activity in the baseline period, highest power in the alpha band, and GMFP reduction in both the pre- and post-TMS windows when removed. See Supplementary Fig. S4B for individual subject component topographies that exceeded 28% in this mid latency window.

Within-subject between-site SI was 0.32 ± 0.019, and between-subject SI was very low for these components, 0.10 ± 0.026. These SI values indicated that the mid-latency components did not exhibit high site specificity but did exhibit subject specificity. Time-course of component amplitude peaks between sites suggested these components were not time locked to the TMS pulse.

See Supplementary Table S4 for similarity of mid latency components between-subject and within-subject between-site from all stimulation conditions, and Supplementary Table S5 for SI of TEPs pre- and post-removal of mid-latency components.

## Discussion

In the present study, we show that auditory-evoked potentials (AEPs) evoked by the sound associated with the discharge of each TMS pulse can be isolated and extracted from the TEP after performing ICA on merged active and sham stimulation conditions. Using data from two separate studies, we show effective and conservative extraction of AEP with younger adult as well as an older population, with variations of sham stimulation protocols, and with different TMS devices and EEG systems. Of note, one of the sham stimulation protocols included electrical stimulation of the skin, as this is increasingly considered an important element of an effective sham design^18,77^. We show this method may preserve residual TEP in early, mid and late latency windows. As an exploratory analysis, we evaluated the residual mid latency TEP for specificity of stimulation site, group, and individual. This analysis suggests that the method may reveal non-specific alpha band modulations that were previously obscured by AEP. Our group level analyses of pre- and post-AEP removal suggest that the TEP is composed of transcranial evoked potentials, sensory-evoked potentials, and site-independent modulation of ongoing oscillations.

To our knowledge, our study represents an important and novel advance by rigorously and quantitatively examining the effects on the TEP of removing an AEP component that is shared between active and sham stimulation. Another aspect of our study that is unique is that the two cohorts that were used differed in several important factors, including age, gender, the details of the sham application, and the use of auditory noise masking. The consistency of results across the two cohorts demonstrates the robustness of the AEP removal method to these multiple experimental factors.

### Efficacy and advantages of an ICA-based approach to removing AEP

Options are needed for removing AEP from TEP to reveal other brain responses to TMS. Although earplugs and masking^6,18,33^ can be used to attenuate AEP, and foam padding can reduce bone conduction of the sound^34^, these techniques do not always work as effectively as anticipated. Many groups have observed AEP even after using these techniques^16,17,34–36^. Consistent with this literature, in our older cohort, in which auditory noise masking was used, a majority of participants perceived the ‘click’ and had an AEP. This may be due to contrasting acoustic properties of the ‘click’ sound and noise masking, or to multisensory contributions to auditory perception and the AEP. However, regardless of the reason for the persistent AEP, options for effective handling of the AEP after data collection could be of great use, and we present one option that is effective. Further, we show that this method is also conservative in that it preserves the earliest TEP that is stimulation site specific and reveals later activity that could carry information about subject-specific neural modulations.

We show that removing AEPs using this structured ICA-based process significantly reduced GMFP (whole scalp) and LMFP (of left DLPFC and IPL ROIs) in the post-stimulation TEP (14 to 400 ms), driven by time windows consistent with the N100 and P200 temporal characteristics of AEP. Further, supported by cosine similarity analysis, we show that removing AEPs reduces TEP similarity between-subjects and between stimulation conditions. Similarity is reduced most in a mid-latency window, but nevertheless remains higher than mid-range for the 0–1 SI rating. Residual TEP in this window has a time course and topography unique from AEP, and follow-up analyses suggest this could be alpha modulation that is not stimulation site specific but is unique to individual subject.

One property of our isolated AEP components that might seem counterintuitive is that there are slight differences in amplitude of the AEP between sites (Fig. 1C). The acoustic properties of the ‘click’ were identical across conditions^6,18^, but the AEP amplitudes were not. This could be due to differences between conditions in coil distance from the ears and/or bone conduction. Nikouline et al.^27^ observed an amplitude difference between AEPs when the coil was pressed against the scalp, held 2 cm above the scalp in the air, or 2 cm above the scalp but with the use of a plastic spacer. In that study, all stimulation conditions were over the same left hemisphere M1 target, and time course of the AEPs were comparable. The amplitude of N100- and P180 peaks were smallest when the coil was held above the scalp, intermediate when a spacer was used, and largest when the coil was pressed directly to the scalp, demonstrating how coil distance from scalp and bone conduction can contribute greatly to AEP amplitudes. Due to these results, AEP should not be expected to have comparable peak amplitudes under different stimulation conditions. Consequently, merely subtracting out the mean sham-evoked potential from active stimulation TEPs would not suffice in accounting for differential AEP amplitudes with other stimulation sites. In contrast, one of the strengths of the ICA approach is that as the underlying neural generators (and thus the resulting scalp EEG topographies) are stable, differential activations on distinct trials can be isolated and removed.

One concern is that it is not known what the “ground truth” TEP without any auditory stimulation (including auditory noise masking) should look like. Rocchi et al. showed suppressed AEP with subthreshold stimulation and noise masking^18^, but future work is needed that demonstrates noise masking efficacy for suprathreshold intensities, and the resulting TEPs when adequate noise masking is used. The recently developed TAAC tool^40^ may be particularly helpful in this regard.

To further validate the ICA method, TEPs devoid of AEP should ideally be compared to TEPs remaining after ICA-based removal of AEP. However, it is still worth noting that adequate noise masking volumes may not be achievable (due to safety or tolerability reasons) in all participants, and thus this may not always be possible. Another factor is that it is unknown what effects auditory noise masking itself has on the TEP, and this needs to be examined in future work. Sustained auditory noise can improve sensory perception^44,48^, modulate cortical and sensorimotor excitability^45,46^, and have impacts on cognitive task performance^48^ and, most importantly for the use in TMS, evoked potentials^49–51^. In addition, if noise is presented at a volume that is uncomfortable for participants, there may be modulations in the TEP that reflect a pain related state change^37–39^. A benefit of the ICA-based technique is that is can be applied to data collected without the use of noise masking sounds and can also be applied if noise masking procedures are not fully effective in masking the TMS pulse.

### Classification of AEP components

Features of the AEP time course, topography and presence in sham can be used for identifying and classifying AEP components, based on the literature from TMS-EEG and from other studies of auditory perception^78^.

Because the auditory system is particularly sensitive to timing, this is reflected in temporal consistency of neural responses to sound. Auditory activity can be observed through multiple stages of the ascending auditory pathway with temporal precision, beginning with the auditory brainstem response (ABR; Jewett waves I–VII; 0–10 ms after stimulus onset) and followed by MLRs originating from medial geniculate nucleus and primary auditory cortex (“Mid-latency responses”; 10–60 ms after stimulus onset)^79^. Sensory AEP is generally described 40–200 ms after the stimulus, before P300 and other cognitive components^27–29,80,81^. Because of axonal divergence up the auditory pathway, neuronal population increases and so do component amplitudes, with the N100 and P200 having the largest peak amplitudes. The AEP consists of at least three subcomponents, described as the “P50-N100-P160 complex”^82–84^ and the “P1-N1-P2 complex”^85,86^. We use nomenclature consistent with TMS-EEG literature^21,26^, and describe the subcomponents at 50, 100, and 200 ms of the well-documented sensory AEP. Because of the consistent time course of these subcomponents, with a smaller amplitude peak at 50 ms and larger amplitude peaks at 100 and 200 ms, time course can be used to identify AEP^29^. Our criteria for AEP classification adhered to this time course of three subcomponents at 50, 100 and 200 ms.

Source analyses of AEP subcomponents reveal that P50 originates in primary auditory cortex and N100 and P200 subcomponents originate in surrounding belt areas of A1^29,87,88^. Because of the mirrored, time-locked bilateral sources (left/right auditory cortices), distribution in scalp electrodes is symmetrical and gives the appearance of a single central deep dipole^31,32^. Our criteria for AEP classification included this expected central and symmetrical scalp topography.

Sham TMS protocols that use an active coil should result in a similar or identical AEP response if the acoustic properties of the “click” are the same across sham and active protocols^52^. Assuming the sham protocol is not itself inducing activation of cortex, the AEP response in sham represents non-TMS-evoked potentials. Our criteria for selecting AEP components included a rigid adherence to this assumption—we only classified components as AEP if they were shared across stimulation site and sham stimulation.

### Site-specificity of TMS-evoked potentials and other modulated oscillations

Although there is evidence to support that TEP can have stimulation site specificity^9,23,27,89^, there is accumulating evidence for non-specificity in mid latency and late latency windows^16,17,23,25^. The present data provide support for the notion that the early responses to TMS are highly specific to the site of stimulation, and thus likely represent transcranial-evoked elements. In contrast, we found decreased site-specificity in later time periods, especially in middle latencies (between 77 and 272 ms in the younger group, and between 87 and 285 ms in the older cohort). The cross-site similarity of these mid-latency potentials decreased significantly after removal of the AEP component but remained mid-range and higher than both the earlier and later elements of the TEP.

By analyzing the residual mid-latency components, we found that there is prominent modulation of alpha band oscillation during this time-period, which is present in both active and sham conditions. To our knowledge, alpha modulation exactly like this has not been described previously following non-M1 targets and sham stimulation, although there is some precedent for alpha and beta modulation with single pulse TMS to M1 (^14,15,69^, for reviews^21,90–92^), which has been attributed to cortico-cortical neuronal processing^93–95^, or to sensory return from muscle twitches^69,96,97^. Because the mid-latency components in both cohorts appear to synchronize after the TMS pulse but are not time-locked to the pulse, we describe this activity as non-specific modulated alpha. Notably, this alpha modulation is similar across stimulation sites but distinct across subjects, suggesting that it reflects subject-specific intrinsic oscillatory dynamics.

Alpha band modulation in the post-TMS period could be caused by some aspect of the stimulation that may not be transcranially evoked. The TEP recording in this window includes transcranial activity, sensory potentials, and attentional and behavioral responses to stimulation events. It is outside the scope of this paper to define all modulated oscillatory dynamics in the post-TMS period. However, briefly here we speculate about some explanations for the mid-latency components that were revealed with the presented AEP-removal method. One possibility is endogenous alpha phase resetting, due to subject specific inhibitory responses to activation of cortical circuits, both transcranially and sensory-evoked. Suppression of cortical networks following TMS, reflected in the N100 time window, may be mediated through GABA-B receptors^98^. Other possible explanations include non-specific modulation of arousal state, micro-blinks or micro-saccades^99–101^, secondary downstream effects of auditory stimulation, predictive auditory processing of repetitive sounds, and other forms of event processing^102–105^.

### Theoretical concerns

All influences of auditory stimulation during an experiment cannot be isolated using these methods and need to be considered carefully in the context of experimental parameters and goals. For example, auditory stimulation can impact aspects of attention^100,101,106^ and motor system excitability^107,108^. Further, if the design captures behavioral response, such as reaction time to a visual stimulus, intersensory facilitation should be accounted for^109–112^. At a neurophysiological level, the ICA technique can be used to suppress the AEP in the primary auditory region. However, the secondary downstream effects may persist (and indeed, may account for the observed alpha synchronization seen in middle latencies even in the sham). In the present study, we describe a method that can be used to examine in detail three dominant contributors to the TEP waveform (transcranial evoked potentials, sensory-evoked potentials, and site-independent modulation of ongoing oscillations), and not all consequences of auditory stimulation. The described technique can be used to study the three dominant parts of the TEP waveform with the caveat that this compartmentalized view of brain network activity may not be ecologically valid due to the multisensory nature of TMS.

### Limitations

One limitation of our approach is that the AEP components were identified and labeled as AEP in a non-automated way. This is a pervasive concern with using ICA for EEG data cleaning—the standard is to classify components by eye using component properties such as time course, topography, and spectral profile. This practice can be time consuming and susceptible to human bias. Many algorithms are useful in identifying specific non-brain artifacts such as eye and muscle, although routinely mislabel other artifacts such as heartbeat. Because AEP is a non-artifact brain component, the component properties can be similar to other neural components and more difficult to distinguish from TMS-evoked neural components. However, unlike other components in the TEP, AEP is very stereotyped in time course and topography and detection can be further aided by the inclusion of a sham stimulation block.

With use of the above-described time course, topographical and condition in-specific features, robust and stereotyped AEPs can easily be identified, as demonstrated in Fig. 1. To expedite data processing and limit human rater bias, we suggest an automated AEP component identification is needed that classifies the AEP based on these stereotyped features^78^.

A second potential limitation is that there could be a cost of analyzing the TEP with the AEP removed if condition, population, or individual participant relevant information is being carried in the auditory response. The auditory response may be relevant for questions related to auditory cortical excitability, plasticity, and excitability gain control. However, because the components removed are likely to be predominantly AEP-related, they can be compared post-hoc across conditions or between populations or individual participants.

Importantly, although AEP was identified using strict criteria based on the TMS-EEG and auditory perception literature, it is likely the AEP described is not well characterized as purely auditory in nature. In fact, somatosensory^19^ and multimodal^41–43^ perception can evoke a central N100-P200 complex, sometimes referred to as the vertex potential (VP). The VP is susceptible to perceived saliency and attention^19^, illusory perception^41^, and can induce modulation of motor excitability^20^. Using our criteria, it would be impossible to differentiate between unimodal auditory AEP and somatosensory or multimodal VP responses during the perceptual experience of TMS. VP time-course and topography matches that of our identified AEP, and may fundamentally reflect the same cortical process.

However, because the AEP potential classified in this work was shared between active and sham stimulation conditions, we believe it to be sensory in nature and not perturbation specific (i.e., not transcranially-evoked cortical responses to TMS). Regardless of the specific nature of this sensory potential, the ICA-based approach described here allows for identification of the potential and isolation from TMS-evoked cortical activity. We suggest that adoption by TMS-EEG researchers of the more accurate nomenclature “vertex potential/VP” may be useful in future investigations of the sensory potential and experimental treatments to reduce the impact of the sensory EEG complex on TEP.

A growing literature suggests that N100/P180 reflects changes in motor cortical excitability^10,98^. These works may describe TMS-evoked activity in the same time window as the vertex N100-P200 described here, particularly if shown to contrast with sham conditions. Proper control conditions are necessary for distinguishing between TMS-evoked and sensory contributors to the TEP. One possibility, particularly if the noise masking procedure in those studies did not entirely attenuate the AEP and/or if a somatosensory activation was present, is that these TMS-evoked elements in the N100/P180 time range actually reflect the vertex potential related to residual sensory elements, and that the vertex potential itself may be modulated by pharmacological manipulations.

### Implications

TMS is increasingly recognized as having significant potential utility in the neurophysiologic characterization of neurological disorders, as well as in the characterization of typical mechanisms of network connectivity and plasticity. However, due to the presence of sensory-evoked potentials such as the AEP, adequate experimental designs as well as appropriate and effective preprocessing of the TEP are critical. Consistent with other recent studies, our results suggest that early-latency TEPs are stimulation site specific and largely unaffected by the AEP, and therefore could be optimal for characterization of evoked responses across groups or stimulation conditions. However, later latencies in the TEP are more at risk for sensory contamination, and therefore effective suppression or isolation is needed. With adequate preprocessing, later components of the TEP show features that are stable across time, specific to the individual, and may be relevant for cognitive mechanisms; future investigations are needed to further characterize the properties of these later components of the TEP. In addition, the factors that affect AEP topography, time-course and amplitude could be studied to better understand the condition, population, or individual participant information that is carried in the auditory response.

## Supplementary Information

Supplementary Information.

## Abbreviations

BIDMC: Beth Israel Deaconess Medical Center
BWH: Brigham and Women’s Hospital
HMS: Harvard Medical School
HSL: Hebrew SeniorLife
MGH: Massachusetts General Hospital
PI: Principal investigator
UCONN: University of Connecticut Health Center
