# Supplementary material for: A structured ICA-based process for removing auditory evoked potentials
Source: Sci Rep. 2022 Jan 26;12:1391. doi: 10.1038/s41598-022-05397-3 (PMC8791940; doi:10.1038/s41598-022-05397-3)
Supplement: Supplementary file 1 — Supplementary Information. [file 41598_2022_5397_MOESM1_ESM.pdf]

Supplementary

Non-parametric permutation based suprathreshold cluster size analysis: This analysis was performed on GMFP of the sham condition, following [73]. N clusters were identified that are greater than baseline (at alpha level of 0.01) for all consecutive samples in at least 5 ms. Cluster sums were calculated for each of those n clusters. Pre (-400 to -14 ms) and post-TMS (14 to 400 ms) time courses were shuffled 1000 times, and 95% and 99% cluster-sum values were calculated in each shuffled dataset. In the un-shuffled dataset, clusters greater than the 99th percentile were identified. Percent variance (pvaf) was calculated for each identified cluster. We removed the peak pvaf contributor, and then reassessed cluster sums, iteratively with the notion that eventually all clusters significantly greater than baseline would be removed.

In the young adult cohort, after four iterations, it became apparent that removing components in this way was reducing GMFP in the baseline window as well as in the post-TMS window, resulting in new significant clusters with more iterations. We found that the components identified in the first or first and second iterations were the same components identified using the percent variance threshold for components contributing most to the mid latency window, described above. In the older cohort, we used the same cluster size analysis, removing components in four iterations. We found that removing these components reduced GMFP in the baseline window as well as in the post-TMS window, resulting in new significant clusters with more iterations.

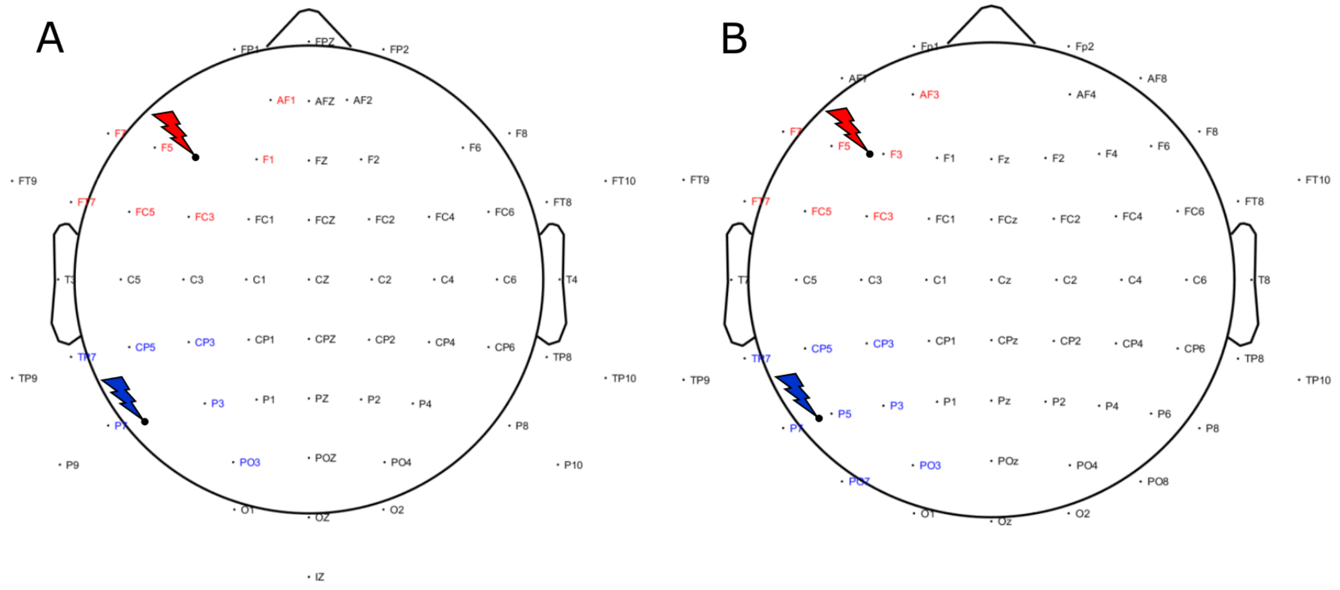

Figure S1. Electrode arrays used with the young adult cohort (A) and the older SAGES cohort (B). Electrodes are color coded to indicate ROI used in the LMFP analysis for L DLPFC (red) and L IPL (blue).

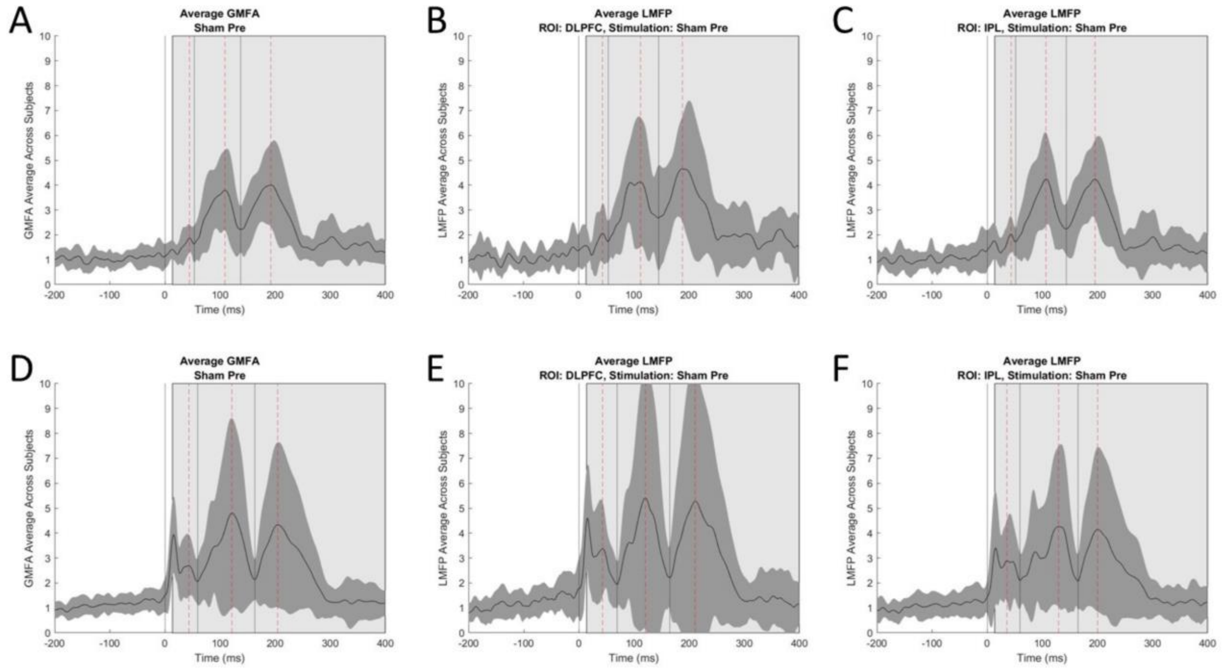

Figure S2. Time windows used to assess reduction in GMFP and LMFP AUC in time ranges corresponding to the three subcomponents of the Auditory Evoked Potential (AEP). Windows were defined in each study cohort for GMFP, LMFP of the DLPFC ROI, and LMFP of the IPL ROI, using latency of the minimum amplitude between peaks (amplitudes greater than two standard deviations above baseline) in the sham stimulation condition. Sham stimulation pre-AEP removal peaks (red lines) and troughs (black) used to define windows in the young adult cohort for GMFP (A), DLPFC ROI (B), and IPL ROI (C), and in the older SAGES cohort for GMFP (D), DLPFC ROI (E), and IPL ROI (F).

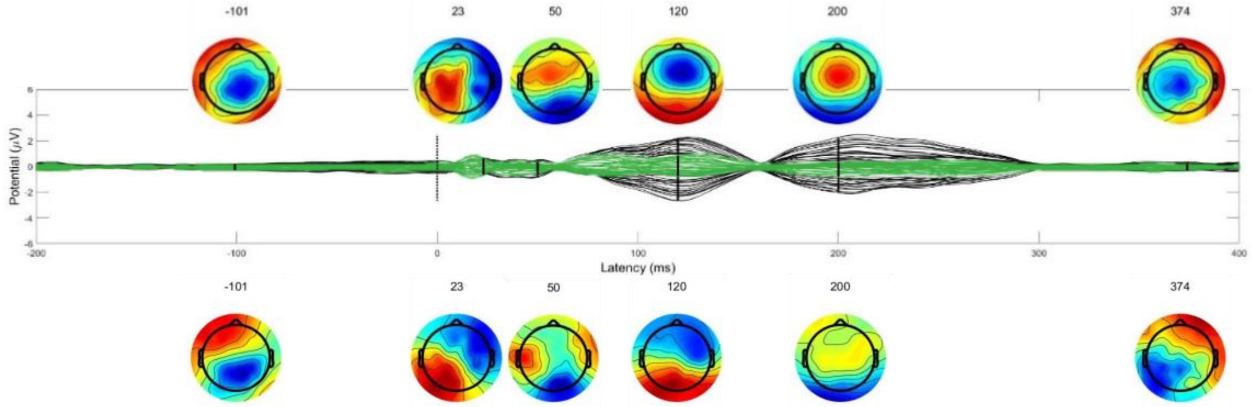

Figure S3. Sham condition TEPs/topography pre (black/above) and post (blue/below) removal of AEP components. Topoplots are greyscale if no peak is present at the indicated latency.

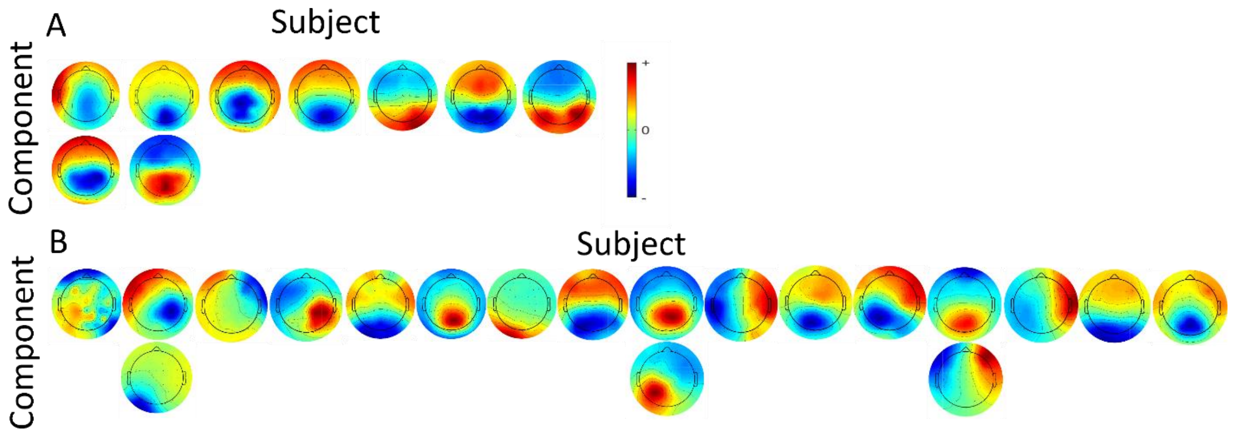

Figure S4. Components contributing the highest percent variance in the mid latency window, determined using a pvaf threshold of 28% in the (A) young adult cohort and (B) older cohort.

**SI of AEP**		Cohort 1				Cohort 2 (Older)			
Extracted from -->		IPL, DLPFC	Sham, DLPFC	Sham, IPL	Average	IPL, DLPFC	Sham, DLPFC	Sham, IPL	Average
Btw. Site	Mean	0.86	0.81	0.83	0.83	0.68	0.65	0.64	0.66
	Std. Err.	0.03	0.03	0.06	0.01	0.07	0.06	0.05	0.01
Extracted from -->		DLPFC	IPL	Sham	Average	DLPFC	IPL	Sham	Average
Btw. Subj.	Mean	0.49	0.53	0.47	0.49	0.42	0.35	0.31	0.36
	Std. Err.	0.03	0.03	0.03	0.02	0.02	0.02	0.02	0.03

Table S1. Similarity of AEP components extracted from each stimulation condition. Similarity index averages and standard error for all between-site comparisons and within-site between-subject comparisons of the removed AEP components.

		Cohort 1			Cohort 2 (Older)		
**GMFA**		Pre/Post	Stim. Site	Interaction	Pre/Post	Stim. Site	Interaction
Full TEP	F Stat	21.78	8.61	2.28	29.27	7.75	6.04
	P-Val	0.001	0.002	0.13	<0.0001	0.002	0.005
P50	F Stat	< 0.0001	10.90	1.41	1.18	2.34	0.41
	P-Val	0.99	0.001	0.27	0.29	0.11	0.67
N100	F Stat	9.14	7.89	0.16	21.08	4.16	6.63
	P-Val	0.014	0.003	0.86	<0.0001	0.023	0.003
P200	F Stat	20.49	6.87	5.65	31.26	9.72	4.58
	P-Val	0.001	0.006	0.012	<0.0001	<0.0001	0.017
**LMFA DLPFC**							
Full TEP	F Stat	13.41	5.64	1.73	18.35	5.89	5.99
	P-Val	0.005	0.013	0.21	<0.0001	0.006	0.006
P50	F Stat	0.47	7.17	2.57	0.014	0.77	0.42
	P-Val	0.51	0.005	0.10	0.91	0.47	0.66
N100	F Stat	6.61	4.86	0.75	19.50	4.22	5.37
	P-Val	0.030	0.021	0.49	<0.0001	0.022	0.009
P200	F Stat	17.93	3.02	2.73	15.42	7.42	3.32
	P-Val	0.002	0.074	0.092	0.001	0.002	0.047
**LMFA IPL**							
Full TEP	F Stat	27.70	7.28	2.46	16.89	10.43	4.55
	P-Val	0.001	0.005	0.11	0.001	<0.0001	0.017
P50	F Stat	0.090	4.43	0.31	1.08	3.28	0.029
	P-Val	0.77	0.027	0.74	0.31	0.048	0.97
N100	F Stat	4.58	3.73	0.041	16.69	3.24	4.41
	P-Val	0.061	0.044	0.96	0.001	0.050	0.019
P200	F Stat	26.69	8.92	5.92	13.49	12.29	2.73
	P-Val	0.001	0.002	0.011	0.002	<0.0001	0.078

Sig. < .01

Sig. < .05

Sig. < .004

Table S2. F-statistics and p-values from 2 (version: pre/post-AEP removal) by 3 (stimulation site: DLPFC/IPL/sham) repeated measures ANOVAs of global and local mean field potential area under the curve for the full TEP (14-400 ms), and in three windows corresponding by the three subcomponents of the AEP (P50, N100, P200), defined using sham condition peak/trough analysis.

		Cohort 1								Cohort 2 (Older)							
		DLPFC		IPL		Sham		Average		DLPFC		IPL		Sham		Average	
**Btw. Subj. SI**		Pre	Post	Pre	Post	Pre	Post	Pre	Post	Pre	Post	Pre	Post	Pre	Post	Pre	Post
Full TEP	Mean	0.48	0.14	0.50	0.14	0.46	0.18	0.48	0.15	0.42	0.18	0.39	0.19	0.30	0.13	0.37	0.16
	Std. Err.	0.02	0.03	0.02	0.02	0.02	0.03	0.01	0.01	0.01	0.01	0.01	0.01	0.02	0.01	0.04	0.02
	T-Stat							17.83								5.03	
	P-Value							0.000029								0.0037	

		IPL, DLPFC		Sham, DLPFC		Sham, IPL		Average		IPL, DLPFC		Sham, DLPFC		Sham, IPL		Average	
**Btw. Site SI**		Pre	Post	Pre	Post	Pre	Post	Pre	Post	Pre	Post	Pre	Post	Pre	Post	Pre	Post
Full TEP	Mean	0.74	0.50	0.68	0.44	0.72	0.46	0.71	0.47	0.63	0.41	0.58	0.39	0.53	0.32	0.58	0.37
	Std. Err.	0.05	0.05	0.05	0.04	0.04	0.05	0.02	0.02	0.05	0.04	0.06	0.05	0.05	0.05	0.03	0.03
	T-Stat							9.17								5.29	
	P-Value							0.000029								0.0031	
Early	Mean	0.29	0.17	0.33	0.19	0.54	0.35	0.39	0.24	0.30	0.18	0.28	0.26	0.23	0.13	0.27	0.19
	Std. Err.	0.12	0.09	0.12	0.11	0.07	0.07	0.08	0.06	0.07	0.05	0.07	0.06	0.07	0.07	0.02	0.04
	T-Stat							1.61								1.98	
	P-Value							0.092								0.060	
Mid	Mean	0.84	0.64	0.77	0.51	0.82	0.55	0.81	0.57	0.77	0.59	0.71	0.49	0.67	0.45	0.72	0.51
	Std. Err.	0.04	0.05	0.04	0.04	0.03	0.07	0.02	0.04	0.04	0.05	0.04	0.05	0.05	0.05	0.03	0.04
	T-Stat							5.68								4.17	
	P-Value							0.0024								0.0070	
Late	Mean	0.51	0.40	0.40	0.36	0.34	0.28	0.42	0.35	0.27	0.24	0.21	0.17	0.24	0.23	0.24	0.21
	Std. Err.	0.09	0.11	0.12	0.11	0.09	0.06	0.05	0.03	0.07	0.07	0.06	0.07	0.08	0.08	0.02	0.02
	T-Stat							1.15								1.00	
	P-Value							0.16								0.19	

Sig. < .01

Sig. < .05

Table S3. Similarity index averages and standard error for all between-site comparisons and within-site between-subject comparisons of TEPs pre- and post-AEP removal.

**SI of Mid Latency ICs**		Cohort 1				Cohort 2 (Older)			
Extracted from -->		IPL, DLPFC	Sham, DLPFC	Sham, IPL	Average	IPL, DLPFC	Sham, DLPFC	Sham, IPL	Average
Btw. Site	Mean	0.59	0.46	0.53	0.53	0.35	0.34	0.29	0.33
	Std. Err.	0.04	0.07	0.09	0.04	0.07	0.06	0.07	0.02

Extracted from -->		DLPFC	IPL	Sham	Average	DLPFC	IPL	Sham	Average
Btw. Subj.	Mean	0.08	0.18	0.09	0.12	0.08	0.15	0.08	0.10
	Std. Err.	0.05	0.06	0.06	0.03	0.02	0.02	0.02	0.03

Table S4. Similarity of residual mid latency components identified in each stimulation condition. Similarity index averages and standard error for all between-site comparisons and within-site between-subject comparisons of the residual mid latency components.

		Cohort 1								Cohort 2 (Older)							
		DLPFC		IPL		Sham		Average		DLPFC		IPL		Sham		Average	
**Btw. Subj. SI**		Pre	Post	Pre	Post	Pre	Post	Pre	Post	Pre	Post	Pre	Post	Pre	Post	Pre	Post
Full TEP	Mean	0.14	0.11	0.14	0.08	0.18	0.14	0.15	0.11	0.18	0.14	0.19	0.09	0.13	0.08	0.16	0.11
	Std. Err.	0.03	0.02	0.02	0.02	0.03	0.02	0.01	0.02	0.01	0.01	0.01	0.01	0.01	0.01	0.02	0.02
	T-Stat							1.89								2.21	
	P-Value							0.066								0.046	

		IPL, DLPFC		Sham, DLPFC		Sham, IPL		Average		IPL, DLPFC		Sham, DLPFC		Sham, IPL		Average	
**Btw. Site SI**		Pre	Post	Pre	Post	Pre	Post	Pre	Post	Pre	Post	Pre	Post	Pre	Post	Pre	Post
Full TEP	Mean	0.50	0.44	0.44	0.38	0.46	0.44	0.47	0.42	0.41	0.41	0.39	0.36	0.32	0.29	0.37	0.35
	Std. Err.	0.05	0.07	0.04	0.06	0.05	0.04	0.02	0.02	0.04	0.05	0.05	0.06	0.05	0.06	0.03	0.04
	T-Stat							1.85								0.48	
	P-Value							0.069								0.33	
Early	Mean	0.17	0.14	0.19	0.22	0.35	0.30	0.24	0.22	0.18	0.17	0.26	0.22	0.13	0.14	0.19	0.18
	Std. Err.	0.09	0.07	0.11	0.07	0.07	0.08	0.06	0.05	0.05	0.06	0.06	0.07	0.07	0.07	0.04	0.02
	T-Stat							0.24								0.20	
	P-Value							0.41								0.42	
Mid	Mean	0.64	0.55	0.51	0.44	0.55	0.54	0.57	0.51	0.59	0.57	0.49	0.46	0.45	0.41	0.51	0.48
	Std. Err.	0.05	0.06	0.04	0.07	0.07	0.04	0.04	0.03	0.05	0.04	0.05	0.06	0.05	0.05	0.04	0.05
	T-Stat							1.14								0.45	
	P-Value							0.16								0.34	
Late	Mean	0.40	0.50	0.36	0.38	0.28	0.32	0.35	0.40	0.24	0.26	0.17	0.22	0.23	0.25	0.21	0.24
	Std. Err.	0.11	0.08	0.11	0.07	0.06	0.06	0.03	0.05	0.07	0.07	0.07	0.07	0.08	0.07	0.02	0.01
	T-Stat							-0.83								-1.12	
	P-Value							0.23								0.16	

Sig. < .01

Sig. < .05

Table S5. Mid latency components similarity averages and standard error for all between-site comparisons and within-site between-subject comparisons pre- and post- removal of mid latency components.
